# Risk factors for the development of premature ventricular complex-induced cardiomyopathy: a systematic review and meta-analysis

**DOI:** 10.1007/s10840-022-01421-8

**Published:** 2022-11-21

**Authors:** Jeanne du Fay de Lavallaz, Julien Mézier, Lara Mertz, Diego Mannhart, Teodor Serban, Sven Knecht, Qurrat-ul-ain Abid, Tai Tri Nguyen, Michael Kühne, Christian Sticherling, Henry Huang, Michael R. Gold, Patrick Badertscher

**Affiliations:** 1 Cardiovascular Research Institute Basel and Department of Cardiology, University Hospital Basel, Spitalstrasse 2, 4056 Basel, Switzerland; 2 Department of Cardiology, Rush University Medical Center, Chicago, IL USA; 3 Department of Medicine, Cardiology Division, Medical University of South Carolina, Charleston, SC USA

**Keywords:** Premature ventricular contractions, Ventricular arrhythmias, PVC-induced cardiomyopathy, Hear failure

## Abstract

**Background:**

Premature ventricular complexes (PVCs) are a potentially reversible cause of heart failure. However, the characteristics of patients most likely to develop impaired left ventricular function are unclear. Hence, the objective of this study is to systematically assess risk factors for the development of PVC-induced cardiomyopathy.

**Methods:**

We performed a structured database search of the scientific literature for studies investigating risk factors for the development of PVC-induced cardiomyopathy (PVC-CM). We investigated the reporting of PVC-CM risk factors (RF) and assessed the comparative association of the different RF using random-effect meta-analysis.

**Results:**

A total of 26 studies (9 prospective and 17 retrospective studies) involving 16,764,641 patients were analyzed (mean age 55 years, 58% women, mean PVC burden 17%). Eleven RF were suitable for quantitative analysis (≥ 3 occurrences in multivariable model assessing a binary change in left ventricular (LV) function). Among these, age (OR 1.02 per increase in the year of age, 95% CI [1.01, 1.02]), the presence of symptoms (OR 0.18, 95% CI [0.05, 0.64]), non-sustained ventricular tachycardias (VT) (OR 3.01, 95% CI [1.39, 6.50]), LV origin (OR 2.20, 95% CI [1.14, 4.23]), epicardial origin (OR 4.72, 95% CI [1.81, 12.34]), the presence of interpolation (OR 4.93, 95% CI [1.66, 14.69]), PVC duration (OR 1.05 per ms increase in QRS-PVC duration [1.004; 1.096]), and PVC burden (OR 1.06, 95% CI [1.04, 1.08]) were all significantly associated with PVC-CM.

**Conclusions:**

In this meta-analysis, the most consistent risk factors for PVC-CM were age, non-sustained VT, LV, epicardial origin, interpolation, and PVC burden, whereas the presence of symptoms significantly reduced the risk. These findings help tailor stringent follow-up of patients presenting with frequent PVCs and normal LV function.

**Supplementary Information:**

The online version contains supplementary material available at 10.1007/s10840-022-01421-8.

## Introduction

Premature ventricular complex-induced cardiomyopathy (PVC-CM) is defined as the development of left ventricular dysfunction (left ventricular ejection fraction (LVEF) of < 50%) caused solely by frequent PVCs [1]. Superimposed PVC-CM can be defined as worsening of LVEF by at least 10% due to frequent PVCs in a previously known CM [1]. Currently, diagnosis of PVC-induced CM can only be made during follow-up, by showing documentation of complete LVEF recovery in absence of PVCs after successful treatment [2].

Clinical studies have found that a high PVC burden is associated with an increased risk of systolic heart failure (HF) (hazard ratio [HR]: 1.48 to 1.8) [3, 4]. Two main studies have shown that PVC burden > 16% and 24% best identifies patients with a diagnosis of PVC-CM [5, 6]. Nevertheless, some patients do not develop CM even with a high PVC burden, whereas other patients develop CM with a burden as low as 6% [7]. Thus, it is likely that other patients’ characteristics and/or PVC features besides PVC burden play a role in the pathophysiology of PVC-CM. Multiple predictors of PVC-CM were described including male sex, lack of symptoms or duration of palpitations [8], variability of PVC coupling interval (dispersion) [9], interpolation of PVCs [10], QRS duration of PVC > 150 ms [11], or epicardial origin [12].

Prior studies investigating risk factors for PVC-CM were retrospective and were not designed with the main objective of assessing these RFs [3–8, 11, 12]. In addition, the assessed study populations were very heterogeneous and often the main endpoint was not defined with enough precision. Thus, most predictors have been variably reported and further validation is required.

We therefore conducted a systematic review and meta-analysis of studies addressing clinical, ECG, Holter, or echocardiographic risk factors able to differentiate patients having a PVC-induced CM from other forms of CM.

## Methods

This systematic review and meta-analysis received approval from the ethics committee and was registered on PROSPERO (CRD42021243622). The reporting of our results was done according to the PRISMA statement about systematic reviews and meta-analyses [13] (Supplemental Table 1) and followed the latest guidelines about reporting systematic reviews and meta-analyses of prognostic factors studies [14].

### Data sources and search

A comprehensive systematic search was conducted in PubMed, MEDLINE, and Embase by combining keywords synonyms of PVC, heart failure, and risk factors as detailed in the Supplemental appendix. The study registry Clinicaltrial.gov was manually searched using the same terms. The search was conducted once on February 27, 2021, accounting for all articles published between January 1, 2000, and February 27, 2021.

### Study selection

Studies that met the following pre-specified criteria were included: (1) RCTs, prospective, or retrospective observational studies and registers; (2) with at least 50 patients total (with and without PVC-CM); (3) assessing adult patients with at least part of the cohort diagnosed with PVC-CM and at least part of the cohort presenting with PVCs; (4) investigating risk factors for the development of PVC-CM (which were not defined beforehand); (5) reporting summary statistics such as regression coefficients, odds ratios (OR) or HR; (6) assessing the incidence, prevalence, or recovery of heart failure thought to be related to PVCs or the change in ejection fraction (EF) due to the presence, increase, or reduction of PVCs; (7) providing either time-to-event data or cross-sectional data; and (8) providing at least one adjusted (multivariable) risk-factor model.

### Endpoints

The primary endpoint of this meta-analysis was the quantitative meta-analysis of risk factors for the development of PVC-CM. We pre-defined that risk factors should be reported in at least 3 different studies with a compatible definition in order to allow for a meaningful quantitative summary.

Secondary endpoints were either the qualitative analysis of risk factors reported in ≥ 3 different studies or important study characteristics, such as (1) the prevalence of comprehensive work-up to ensure patients diagnosed with PVC-CM did not present with another cause for heart failure; (2) the differences in the reported definitions of PVC-CM; and (3) the assessment of study quality using the validated QUIPS (Quality in Prognosis Studies) tool [15].

### Primary outcome

The primary outcome of this meta-analysis was the presence of PVC-CM, which we pre-defined either as the development, presence, or recovery from heart failure with reduced ejection fraction (HFrEF) in patients with CMP in whom no other cause of heart failure was evident. Further details are available in the supplemental.

### Analysis of risk factors

A meta-analysis was conducted on risk factors presenting ≥ 3 times throughout the studies. When continuous risk factors were presented using cutoffs, the exposure per group (above and below the respective cutoff) was derived as recommended in previous dose-exposure meta-analyses and corresponding guidelines [16–19].

Further details regarding the analysis of risk factors are given in the supplemental.

### Assessment of study quality

Study quality was assessed according to the QUIPS tool [15] and summarized graphically.

### Statistical analysis

The analysis was performed according to the recommendations of the Cochrane Collaboration [20] and the reporting was in line with the Preferred Reporting Items for Systematic Reviews and Meta-Analysis (PRISMA) statement [13] and according to recent guidelines on the conduction of review and meta-analyses of prognostic factor studies [14].

We recorded quantitative measures of baseline characteristics as mean with standard deviation (SD) or median with interquartile range (IQR). To allow for quantitative summaries, we transformed the median with IQR into mean with SDs using a mathematical transformation as proposed in previous research [21].

For the main analysis, in order to increase the number of studies available for the quantitative summary of each risk factor, we summarized odds ratios and hazard ratios as a common measure of risk ratio, as it has been conducted in previous meta-analyses [16, 22].

To allow for the expected heterogeneity in effect measures across studies, summary relative risk estimates and their 95% CIs were estimated from a random effect model [23] that used the inverse variance method as proposed by the metagen package [24], which considers both within- and between-study variation. To estimate the between-study variance, the Tau estimator was calculated according to the DerSimonian-Laird estimator [23, 25]. Statistical heterogeneity among studies was evaluated using the *I*^2^ statistic [26].

Details of the dose–response analysis are available in the supplemental.

Significant heterogeneity was defined as an *I*^2^ statistic of > 50%.

Evidence for publication bias was assessed for PVC burden graphically using contour-enhanced funnel plots [27] and the Egger test.

The risk of bias within each study was assessed using the QUIPS tool.

All statistical analyses were performed using the Statistical Software “R” (R Foundation for Statistical Computing, Vienna, Austria). *P* values < 0.05 were considered as significant.

## Results

### Selected studies

**Fig. 1:**
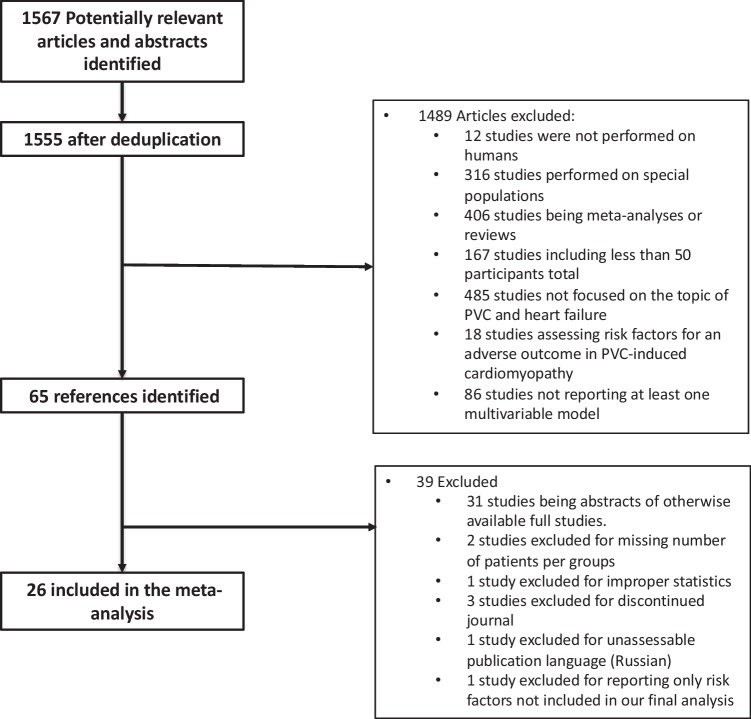
Study selection chart flow

A total of 1567 studies were identified and 1540 were excluded. There were 65 full-text publications reviewed, of which 39 were excluded: 31 studies were based on the same cohorts (mostly representing abstracts of otherwise available complete studies) and 8 studies did not provide risk factors of interest or appropriate statistics. This resulted in 26 studies included in the present systematic review and meta-analysis [5, 7–12, 28–46] (Fig. 1).

Baseline study characteristics are presented in Table 1. The included studies reported data on patients treated between 1989 and 2019. They consisted of 9 prospective and 17 retrospective studies. One of the retrospective studies was a re-analysis of a register (the California Health Care Cost and Utilization Project (CHCCUP)) evaluating 16,757,903 patients that was qualitatively analyzed but was eventually excluded from the meta-analysis because of the bias caused by its extreme weight. The 25 other studies provided a total of 6738 patients.

**Table 1:** Study baseline characteristics

Study number	Main author	Abstract or full study	Study begin	Study end	Study duration (years)	Type	Country	Centers	Number of patients recruited	Number of patient analyzed	Is this a re-analysis of a previous trial?	Previous trial
1	Altıntaş	Full study	2019–01-01	2019–05-01	0.3	Prospective study: cohort study	Turkey	Multicentric	341	341	No	
2	Sadron	Full study	2003–01-01	2012–01-01	9.0	Retrospective study: case–control study	International	Multicentric	168	168	No	
3	Lee	Full study	2011–01-01	2017–01-01	6.0	Retrospective study: cohort study	Australia	Unknown	152	152	No	
4	Penela	Full study				Prospective study: cohort study	International	Multicentric	70	70	No	
5	Park	Full study	2000–01-01	2015–07-01	15.5	Retrospective study: cohort study	Japan	Multicentric	801	180	No	
6	Agarwal	Full study	2005–01-01	2009–12-31	5.0	Retrospective study: cohort study	USA	Multicentric	16,800,000	16,757,903	Yes	California Healthcare Cost and Utilization Project
7	Dukes	Full study	1989–01-01	2000–01-01	11.0	Prospective study: cohort study	USA	Multicentric	1429	1139	Yes	Cardiovascular Health Study
8	Ban	Full study				Prospective study: cohort study	Korea	Monocentric	127	127	No	
9	Yokokawa	Full study				Prospective study: cohort study	USA	Monocentric	315	294	No	
10	Yokokawa	Full study	1999–04-01	2010–12-31	11.8	Retrospective study: cohort study	USA	Monocentric	241	241	No	
11	Baman	Full study				Retrospective study: cohort study	USA	Monocentric	174	174	No	
12	Kanei	Full study	2001–01-01	2006–08-30	5.7	Retrospective study: cohort study	Japan	Monocentric	429	108	No	
13	Kawamura	Full study	2007–01-01	2013–08-30	6.7	Retrospective study: cohort study	USA	Monocentric	214	214	No	
14	Mountantonakis	Full study				Retrospective study: cohort study	USA	Monocentric	69	69	No	
15	Olgun	Full study				Retrospective study: cohort study	USA	Monocentric	51	51	No	
16	Yokokawa	Abstract				Prospective study: cohort study	USA	Monocentric	197	197	No	
17	Blaye-Félice	Abstract				Prospective study: case–control study	International	Multicentric	168	168	No	
18	Yang	Full study	2005–06-28	2013–06-18	8.0	Prospective study: case–control study	USA	Multicentric	5289	264	No	
19	Latchamsetty	Full study	2004–01-01	2013–01-01	9.0	Retrospective study: cohort study	International	Multicentric	1185	1185	No	
20	Voskoboinik	Full study	2012–01-01	2019–10-01	7.7	Retrospective study: cohort study	International	Multicentric	206	206	No	
21	Azizi	Abstract	2011–01-01	2017–01-01	6.0	Prospective study: case–control study		Monocentric	204	130	No	
22	Yamada	Full study	2010–01-01	2015–01-01	5.0	Retrospective study: case–control study	International	Multicentric	130	130	No	
23	Hamon	Full study	2011–05-01	2013–06-01	2.1	Retrospective study: cohort study	International	Multicentric	107	107	No	
24	Bas	Full study	2005–11-01	2011–09-01	5.8	Retrospective study: cohort study	USA	Monocentric	107	107	No	
25	Gunda	Full study	2014–11-01	2016–10-01	1.9	Retrospective study: cohort study	USA	Monocentric	846	846	No	
26	Del	Full study	2005–11-01	2008–07-01	2.7	Retrospective study: cohort study	USA	Monocentric	70	70	No	

Further details regarding inclusion and exclusion criteria for each study and definitions of both PVC-CM and PVCs are presented in Supplemental Table 3. Fifteen of 26 (57.7%) studies provided a definition of PVC-CM: the CMP was mostly defined as an LVEF < 50% and 9/26 (34.6%) studies took a time component into account (e.g., normalization or increase in the EF over time). The requirement for LVEF improvement in the PVC-CM definition varied from 10 to 15% in these studies.

### Baseline patient characteristics

Often, several groups were analyzed in each study, which did not always report data for the overall cohort. The analyzed groups are presented in Table 2. In summary, the overall patient population was rather young (weighted mean age of 50.2 years old, 55.0 years old when excluding data from the predominant CHCCUP study) and with a weighted mean PVC burden of 16.5% (not reported in the CHCCUP study). The weighted mean percentage of women in the overall analyzed dataset was 57.6%, which decreased to 44.2% when excluding data from the CHCCUP. In a significant proportion of the studies and reported groups, there was no described attempt to assess for the presence of underlying structural heart disease or this detail was not reported (8/26 studies, Supplemental Tables 4 and 5).

**Table 2:** Baseline characteristics of the patient groups in the 26 selected studies

Study number	Group number	Name	Patient number	Diagnosis of arrhythmia	Diagnosis of HF	PVC-CMP	SHD	Age	LVEF	PVC burden	% women	% men
1	1	Overall cohort	341	All	Part	Some	None	50 ± 6	60 ± 2	10 ± 3	40.8	50.4
2	1	Overall cohort	168	All	Part	Some	Some	55 ± 15	48 ± 15	22 ± 13	38.1	61.9
2	2	PVC-CMP group	96	All	All	Some	Some	53 ± 16	38 ± 10	26 ± 12	26.0	74.0
2	3	Control group without PVC-CMP	72	All	None	None	None	56 ± 15	62 ± 7	17 ± 22	54.2	45.8
3	1	Cardiomyopathy group (LVEF < 50%)	54	All	All	Unknown	Some	59 ± 15	39 ± 3	30 ± 6	25.9	74.1
3	2	Control group with LVEF > 50%	98	All	None	None	None	50 ± 16	59 ± 2	19 ± 5	60.2	39.8
4	1	Overall cohort	70	All	All	Some	Some	58 ± 11	34 ± 9	24 ± 4	17.1	82.9
4	2	Myocardial scar	29	All	All	Some	Some	61 ± 8	35 ± 9	24 ± 4	3.4	96.6
4	3	No myocardial scar	41	All	All	Some	Some	56 ± 12	33 ± 9	26 ± 3	26.8	73.2
5	1	Symptomatic with PVC cardiomyopathy	28	All	All	All	None	52 ± 13	35 ± 8	29 ± 16	25.0	75.0
5	2	Symptomatic without cardiomyopathy	116	All	None	None	None	49 ± 15	59 ± 6	21 ± 15	56.0	44.0
5	3	Asymptomatic with PVC cardiomyopathy	24	All	All	All	None	58 ± 15	34 ± 9	31 ± 10	16.7	83.3
5	4	Asymptomatic without cardiomyopathy	12	All	None	None	None	55 ± 16	56 ± 8	28 ± 12	41.7	58.3
6	1	PVC diagnosis	35,817	All	None	None	None	66 ± 17			48.9	51.1
6	2	No PVC diagnosis	16,722,086	None	None	None	None	50 ± 19			57.7	42.3
7	1	Below or equal to the median of percent PVC	587	Part	None	None	None	70 ± 2			63.7	36.3
7	2	Above the median of percent PVCs	552	All	None	Unknown	None	71 ± 2			51.3	48.7
8	1	LV dysfunction (LVEF < 50%)	28	All	All	Some	Unknown	48 ± 14	44 ± 5	31 ± 11	39.3	60.7
8	2	No LV dysfunction (LVEF > 50%)	99	All	None	Unknown	None	43 ± 13	57 ± 3	22 ± 10	66.7	33.3
9	1	Overall cohort	294	All	Part	Some	None	48 ± NA	52 ± 12	19 ± 14	53.4	46.6
9	2	Reversible PVC-induced cardiomyopathy	113	All	Part	All	None	49 ± 15	40 ± 10	27 ± 12	36.3	63.7
9	3	No PVC-induced cardiomyopathy	181	All	Part	None	None	48 ± 13	60 ± 4	14 ± 12	63.5	36.5
10	1	Cardiomyopathy	76	All	All	All	None	48 ± 16	36 ± 9	28 ± 12	32.9	67.1
10	2	No cardiomyopathy	165	All	Part	None	None	48 ± 13	59 ± 5	15 ± 13	61.2	38.8
11	1	No cardiomyopathy	117	All	Part	None	Unknown	48 ± 12	59 ± 4	14 ± 12	55.6	44.4
11	2	Cardiomyopathy	57	All	All	Some	Unknown	49 ± 12	35 ± 9	33 ± 14	38.6	61.4
12	1	< 1000 PVC/24 h	24	All	Part	Unknown	Unknown	47 ± 16			29.2	20.8
12	2	1000–10,000 PVC/24 h	55	All	Part	Unknown	Unknown	52 ± 17			63.6	36.4
12	3	> 10,000 PVC/24 h	29	All	Part	Unknown	Unknown	48 ± 15			69.0	31.0
13	1	LV dysfunction	51	All	All	Some	Unknown	50 ± 13	42 ± 5	19 ± 6	49.0	51.0
13	2	No LV dysfunction	163	All	None	Unknown	None	46 ± 14	62 ± 9	15 ± 11	60.7	39.3
14	1	Overall cohort	69	All	All	Unknown	None	51 ± 16	35 ± 9		37.7	62.3
14	2	Patients without pre-existing cardiomyopathy	49	All	All	Unknown	None	50 ± 15	37 ± 8		34.7	65.3
14	3	Patients with pre-existing cardiomyopathy	20	All	All	Unknown	None	55 ± 16	28 ± 7		45.0	55.0
15	1	Patients with pre-existing cardiomyopathy	21	All	All	All	None	50 ± 15	37 ± 10	30 ± 11	33.3	66.7
15	2	Patients without pre-existing cardiomyopathy	30	All	None	None	None	47 ± 16	59 ± 7	14 ± 15	40.0	60.0
15	3	Patients with interpolation	20	All	Part	Some	None			28 ± 12		
15	4	Patients without interpolation	31	All	Part	Some	None			15 ± 15		
16	1	Overall cohort	197	All	Part	Some	Unknown	48 ± 14			54.3	45.7
16	2	Reduced LVEF	56	All	Part	All	Unknown			15 ± 13		
16	3	Normal LVEF	141	All	Part	None	Unknown			29 ± 12		
17	1	PVC-CMP group	93	All	None	All	None	58 ± 14			25.8	74.2
17	2	Non PVC-CMP control group		All	None	Unknown	None					
17	3	Overall cohort	168	Part	None	Some	None			27 ± 12		
18	1	High burden PVC group	66	All	Part	Unknown	Some	64 ± 16	53 ± 12		42.4	57.6
18	2	Control group	198	Part	Part	Unknown	Some	58 ± 20	63 ± 10		56.1	43.9
19	1	Overall cohort	1185	Part	None	Some	None	52 ± 15	55 ± 10	20 ± 13	54.9	45.1
20	1	Derivation cohort with PVC patients	206	All	Part	Some	Unknown	65 ± 16	57 ± 12	12 ± 6	38.3	61.7
20	2	First validation cohort with PVC patients		All	None	None	None					
20	3	Second validation cohort with PVC patients	516	All	None	None	None	56 ± 17	63 ± 4	20 ± 10	54.5	45.5
21	1	EF under 50%		All	All	Some	None					
21	2	PVC-CMP	15	All	All	All	None	60 ± 19		32 ± 17	13.3	86.7
21	3	Control group	103	All	None	None	None			15 ± 13		
22	1	PVC-induced cardiomyopathy	25	All	All	All	None	47 ± 13	42 ± 5	24 ± 15	56.0	44.0
22	2	Normal LVEF	105	All	None	None	None	43 ± 12	60 ± 7	15 ± 11	63.8	36.2
23	1	Overall cohort	107	All	Part	Unknown	Some	56 ± 16	48 ± 14	23 ± 12	35.5	64.5
23	2	Epicardial origin	25	All	Part	Unknown	Some	54 ± 14	42 ± 10	25 ± 10	20.0	80.0
23	3	Endocardial origin	82	All	Part	Unknown	Some	56 ± 16	50 ± 15	23 ± 12	41.5	58.5
23	4	With PVC-CMP	58	All	Part	All	Some	56 ± 15	38 ± 9	28 ± 10	22.4	77.6
23	5	Without PVC-CMP	44	All	None	None	None	56 ± 16	62 ± 7	16 ± 10	56.8	43.2
24	1	With CM	43	All	All	All	None	48 ± 16	38 ± 5	28 ± 12	25.6	74.4
24	2	Without CM	64	All	None	None	None	47 ± 13	58 ± 4	20 ± 10	59.4	40.6
25	1	PVC burden < 1%	599	All	Part	Unknown	Unknown		53 ± 9	1 ± 0	13.9	86.1
25	2	PVC burden 1–2.1%	82	All	Part	Unknown	Unknown		50 ± 10	1 ± 0	8.5	91.5
25	3	PVC burden 2.2–4.9%	81	All	Part	Unknown	Unknown		47 ± 14	4 ± 0	7.4	92.6
25	4	PVC burden 5–24%	83	All	Part	Unknown	Unknown		45 ± 14	14 ± 3	4.8	95.2
25	5	LVEF > 50%	331	All	None	None	None			2 ± 4	11.8	88.2
25	6	LVEF 42.5–49.6%	38	All	All	Unknown	Unknown			2 ± 4	2.6	97.4
25	7	LVEF 30–40%	32	All	All	Unknown	Unknown			3 ± 6	12.5	87.5
25	8	LVEF 12.5–27.50%	34	All	All	Unknown	Unknown			6 ± 10	0.0	100.0
26	1	EF < 50%	17	All	All	All	Unknown	42 ± 17	38 ± 9	29 ± 15	41.2	58.8
26	2	EF ≥ 50%	53	All	None	None	None	39 ± 18	59 ± 6	17 ± 14	62.3	37.7

### Assessment of outcomes

Most of the studies assessed the presence of PVC-CM (17/26), the recovery of LVEF after PVC-CM 4/27 (defined as a binary variable), or the worsening of LVEF suspected to be due to PVC-CM 2/27 (also defined as a binary variable). We conducted a pooled analysis for these three outcomes, as these are solely different ways to define a PVC-CM. Studies reporting continuous LVEF change over time (3/26) were rare (Table 3).

**Table 3:** Derived models in the different studies and recorded outcomes and risk factors

Study ID	First author	Uni- vs multivariable	Outcome	Summarized outcome	Type of model	Risk factors assessed
1	Altıntaş	Multivar	LVEF (continuous)	LVEF (continuous)	Linear regression	PVC burden, interpolation, age, sex, PVC type: outflow origin, PVC type: duration, coupling interval, PVC type: morphology, QRS duration
2	Sadron	Univar	Presence of a PVC-induced cardiomyopathy (categorical)	LVEF change	Logistic regression	Coupling interval, QRS duration, PVC burden, sex, PVC type: morphology, age, PVC type: origin, palpitations
2	Sadron	Multivar	Presence of a PVC-induced cardiomyopathy (categorical)	LVEF change	Logistic regression	Coupling interval, QRS duration, PVC burden, sex, PVC type: morphology, age, PVC type: origin, palpitations
3	Lee	Univar	Presence of a PVC-induced cardiomyopathy (categorical)	LVEF change	Logistic regression	Age, sex, coupling interval, PVC type: outflow origin, PVC type: duration, PVC burden, symptoms
3	Lee	Multivar	Presence of a PVC-induced cardiomyopathy (categorical)	LVEF change	Logistic regression	Age, sex, coupling interval, PVC type: outflow origin, PVC type: duration, PVC burden, symptoms
4	Penela	Univar	Recovery of LVEF (categorical)	LVEF change	Logistic regression	Sex, age, EF, PVC burden, PVC type: origin, PVC type: duration, LVED, SHD, PVC type: morphology
5	Park	Univar	Presence of a PVC-induced cardiomyopathy (categorical)	LVEF change	Logistic regression	Sex, PVC type: duration, QRS duration, PVC burden, PVC type: origin
5	Park	Multivar	Presence of a PVC-induced cardiomyopathy (categorical)	LVEF change	Logistic regression	Sex, PVC type: duration, QRS duration, PVC burden, PVC type: origin
6	Agarwal	Multivar	Presence of a PVC-induced cardiomyopathy (categorical)	LVEF change	Cox hazard proportional model	Sex, race, age, PVC burden, HTN, DM, CAD
7	Dukes	Univar	Worsening of LVEF (categorical)	LVEF change	Logistic regression	PVC burden
7	Dukes	Multivar	Worsening of LVEF (categorical)	LVEF change	Logistic regression	PVC burden
8	Ban	Multivar	Presence of a PVC-induced cardiomyopathy (categorical)	LVEF change	Logistic regression	PVC burden, non-sustained VT
9	Yokokawa	Multivar	Presence of a PVC-induced cardiomyopathy (categorical)	LVEF change	Logistic regression	PVC burden, QRS duration, PVC type: origin, sex
10	Yokokawa	Multivar	Presence of a PVC-induced cardiomyopathy (categorical)	LVEF change	Logistic regression	Symptoms, PVC burden
11	Baman	Univar	Presence of a PVC-induced cardiomyopathy (categorical)	LVEF change	Logistic regression	Sex, PVC burden, PVC type: outflow origin, PVC type: morphology, non-sustained VT, PVC type: origin
11	Baman	Multivar	Presence of a PVC-induced cardiomyopathy (categorical)	LVEF change	Logistic regression	Sex, PVC burden, PVC type: outflow origin, PVC type: morphology, non-sustained VT, PVC type: origin
12	Kanei	Multivar	Presence of a PVC-induced cardiomyopathy (categorical)	LVEF change	Logistic regression	Non-sustained VT
13	Kawamura	Multivar	Presence of a PVC-induced cardiomyopathy (categorical)	LVEF change	Logistic regression	Age, coupling interval, PVC burden, QRS duration, other
14	Mountantonakis	Univar	Recovery of LVEF (categorical)	LVEF change	Cox hazard proportional model	Age, SHD, EF, PVC type: morphology
14	Mountantonakis	Multivar	Recovery of LVEF (categorical)	LVEF change	Cox hazard proportional model	Age, SHD, EF, PVC type: morphology
15	Olgun	Univar	Presence of a PVC-induced cardiomyopathy (categorical)	LVEF change	Logistic regression	PVC burden, interpolation, BB
15	Olgun	Multivar	Presence of a PVC-induced cardiomyopathy (categorical)	LVEF change	Logistic regression	PVC burden, interpolation, BB
16	Yokokawa	Multivar	Presence of a PVC-induced cardiomyopathy (categorical)	LVEF change	Cox hazard proportional model	PVC burden
17	Blaye-Félice	Multivar	Presence of a PVC-induced cardiomyopathy (categorical)	LVEF change	Logistic regression	PVC type: morphology, PVC type: outflow origin, PVC type: origin, PVC burden
18	Yang	Multivar	LVEF (continuous)	LVEF (continuous)	Logistic regression	PVC burden, QRS duration
19	Latchamsetty	Multivar	Presence of a PVC-induced cardiomyopathy (categorical)	LVEF change	Logistic regression	Sex, PVC burden, symptoms, age, PVC type: origin, PVC type: outflow origin, CAD, HTN, PVC type: morphology
20	Voskoboinik	Univar	Worsening of LVEF (categorical)	LVEF change	Logistic regression	Non-sustained VT, sex, coupling interval, PVC type: origin, PVC burden, PVC type: duration, CAD, age, HTN, PVC type: morphology
20	Voskoboinik	Multivar	Worsening of LVEF (categorical)	LVEF change	Logistic regression	Non-sustained VT, sex, coupling interval, PVC type: origin, PVC burden, PVC type: duration, CAD, age, HTN, PVC type: morphology
21	Azizi	Multivar	Recovery of LVEF (categorical)	LVEF change	Logistic regression	PVC burden
22	Yamada	Univar	Presence of a PVC-induced cardiomyopathy (categorical)	LVEF change	Logistic regression	PVC burden, QRS duration, non-sustained VT, interpolation, coupling interval, Q wave amplitude in aV_L_
22	Yamada	Multivar	Presence of a PVC-induced cardiomyopathy (categorical)	LVEF change	Logistic regression	PVC burden, QRS duration, non-sustained VT, interpolation, coupling interval, Q wave amplitude in aV_L_
23	Hamon	Multivar	Recovery of LVEF (categorical)	LVEF change	Logistic regression	Sex, SHD, interpolation, coupling interval, PVC type: origin, QRS duration, PVC burden, PVC type: duration, palpitations
24	Bas	Univar	Presence of a PVC-induced cardiomyopathy (categorical)	LVEF change	Logistic regression	Sex, PVC burden, PVC type: morphology, interpolation, symptoms, PVC type: duration
24	Bas	Multivar	Presence of a PVC-induced cardiomyopathy (categorical)	LVEF change	Logistic regression	Sex, PVC burden, PVC type: morphology, interpolation, symptoms, PVC type: duration
25	Gunda	Univar	Presence of a PVC-induced cardiomyopathy (categorical)	LVEF change	Linear regression	PVC burden
25	Gunda	Multivar	Presence of a PVC-induced cardiomyopathy (categorical)	LVEF change	Logistic regression	PVC burden
26	Del	Multivar	LVEF (continuous)	LVEF (continuous)	Linear regression	PVC burden, non-sustained VT, PVC type: duration, PVC type: morphology, palpitations

### Assessed risk factors

Table 4 presents the occurrence of all risk factors throughout the selected studies and the occurrence of reporting which were suitable for quantitative analysis (≥ 3 occurrences in multivariable model assessing a binary change in LV function).

**Table 4:** Candidate risk factors proposed in the 26 studies and their relative occurrence (either overall or in multivariable models assessing a binary change in LVEF—either an improvement, worsening in EF, or the development of a PVC-CMP—suitable for quantitative summary analysis)

Candidate risk factor	Occurrence in selected studies	Occurrence as multivariable model assessing binary change in LV function
PVC burden	24	20
Sex	13	7
PVC type: origin	11	6
Age	10	3
PVC type: morphology	10	3
PVC type: duration	8	4
QRS duration	8	5
Coupling interval	7	3
Non-sustained VT	6	3
Interpolation	5	3
CAD	4	2
HTN	4	2
Symptoms	4	3
EF	3	1
Palpitations	3	2
SHD	3	2
Symptom duration	3	–
Acute successful ablation	2	
PVC burden reduction	2	
Antiarrhythmic drug use	1	
Atrial fibrillation	1	
Beta-blocker therapy	1	
BNP (pg mL^−1^)	1	
Body mass index > 30	1	
Chronic ablation outcome	1	
Coefficient of variation	1	
Coronary artery bypass graft	1	
Coupling interval dispersion	1	
Diabetes mellitus	1	
Duration of palpitations	1	
Fascicular PVC	1	
First-degree family history of sudden death	1	
History of dizziness	1	
History of myocardial infarction	1	
Inferior axis	1	
Left bundle branch block	1	
LVED	1	
Mean creatinine	1	
Myocardial scar (g) in MRI	1	
Peak deflection index	1	
PVC-CMP index	1	
Q wave amplitude in aV_L_	1	
Q wave ratio in leads aV_L_/aV_R_	1	
Race	1	
Residual PVC burden after ablation	1	
Retrograde P wave	1	
Superiorly directed PVC axis	1	

Supplemental table 6 presents the risk factors analyzed by each study. The exact definitions of each risk factor, as provided by the individual studies, are presented in the supplemental.

PVC burden was the most commonly analyzed risk factor (24/26 studies, 20/26 studies for quantitative summary), followed by sex (13/26), PVC origin (11/26), PVC and morphology (10/26), and PVC and QRS duration (each in 8/26 studies). Only few other risk factors (age, coupling interval, non-sustained VTs, interpolation, and the presence of symptoms) were investigated in ≥ 3 studies and suitable for quantitative summary. Further investigated risk factors were baseline LVEF, coupling interval, polymorphic PVCs, and outflow tract origins. These risk factors did not appear often enough (< 3 appearances) or were differently defined, hence not suitable for quantitative summary.

### Quantitative associations of risk factors with PVC-CM

**Fig. 2:**
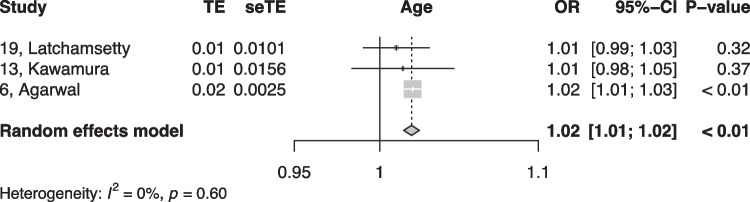
Random effects model showing the overall effect of age on the risk of developing PVC-CM. TE, estimate of treatment effect; seTE, standard error of treatment estimate; OR, odds ratio; CI, confidence interval

**Fig. 3:**
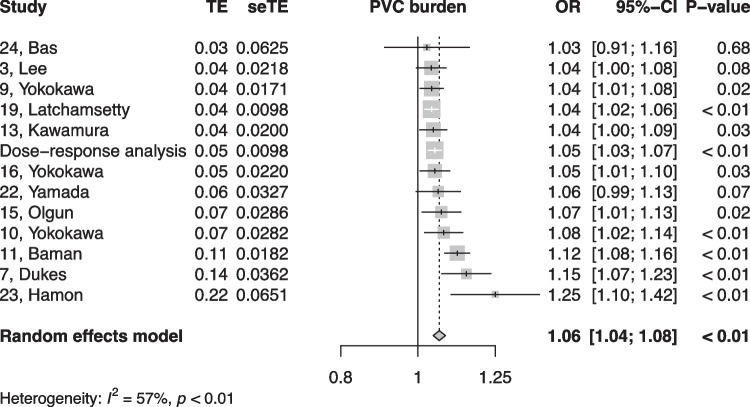
Random effects model showing the overall effect of overall PVC burden on the risk of developing PVC-CM. TE, estimate of treatment effect; seTE, standard error of treatment estimate; OR, odds ratio; CI, confidence interval

**Fig. 4:**
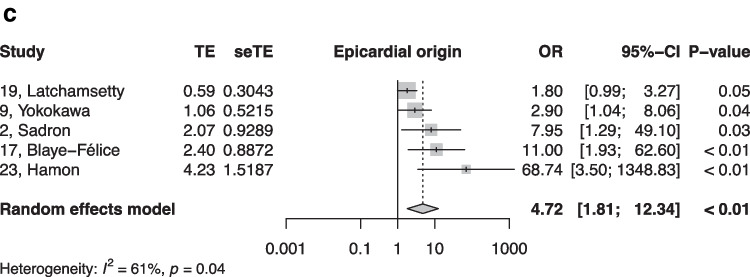
Random effects model showing the overall effect of epicardial origin of the PVC on the risk of the developing PVC-CM. TE, estimate of treatment effect; seTE, standard error of treatment estimate; OR, odds ratio; CI, confidence interval

**Fig. 5:**
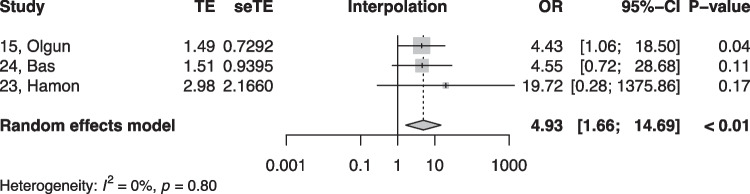
Random effects model showing the overall effect of interpolated PVCs on the risk for PVC-CM. TE, estimate of treatment effect; seTE, standard error of treatment estimate; OR, odds ratio; CI, confidence interval

**Fig. 6:**
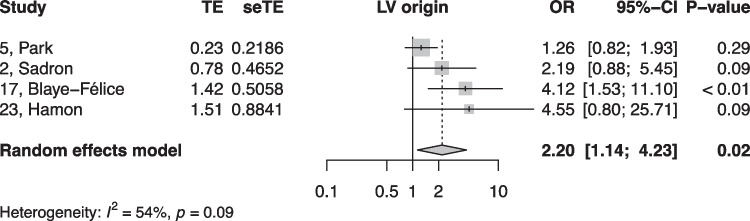
Random effects model showing the overall effect of left ventricular origin of the PVC on the risk of the developing PVC-CM. TE, estimate of treatment effect; seTE, standard error of treatment estimate; OR, odds ratio; CI, confidence interval

**Fig. 7:**
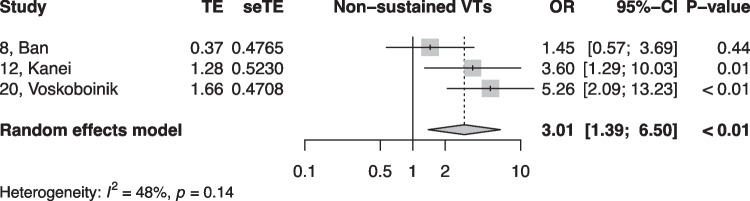
Random effects model showing the overall effect of non-sustained ventricular tachycardia on the risk for PVC-CM. TE, estimate of treatment effect; seTE, standard error of treatment estimate; OR, odds ratio; CI, confidence interval

**Fig. 8:**
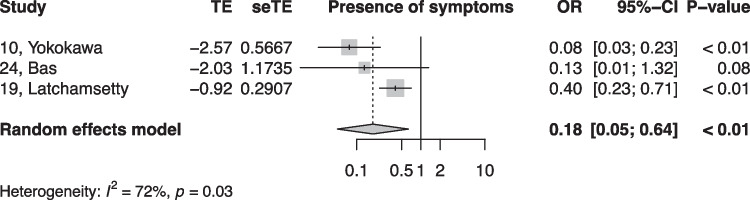
Random effects model showing the overall effect of symptoms on the risk for PVC-CM. TE, estimate of treatment effect; seTE, standard error of treatment estimate; OR, odds ratio; CI, confidence interval

**Fig. 9:**
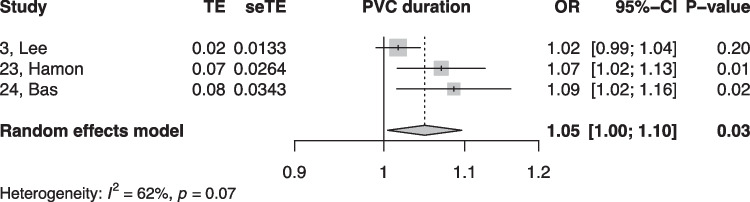
Random effects model showing the effect of PVC duration (per ms increase in QRS PVC duration) on the risk for PVC-CM. TE, estimate of treatment effect; seTE, standard error of treatment estimate; OR, odds ratio; CI, confidence interval

When summarized quantitatively, age (OR 1.02 per increase in year of age, 95% CI [1.01, 1.02]), the presence of symptoms (OR 0.18, 95% CI [0.05, 0.64]), non-sustained VTs (OR 3.01, 95% CI [1.39, 6.50]), LV origin (OR 2.20, 95% CI [1.14, 4.23]), epicardial origin (OR 4.72, 95% CI [1.81, 12.34]), the presence of interpolation (OR 4.93, 95% CI [1.66, 14.69]), PVC burden (OR 1.06 per percent increase in burden, 95% CI [1.04, 1.08]), and PVC duration (OR 1.05 per ms increase in QRS-PVC duration [1.004; 1.096]) were all significantly associated with PVC-CM (Figs. 2, 3, 4, 5, 6, 7, 8, and 9). Coupling interval, polymorphic PVCs, outflow tract origin, sex, and QRS duration did not display a significant association (Supplemental Fig. 1).

### Dose–response analysis of PVC burden

**Fig. 10:**
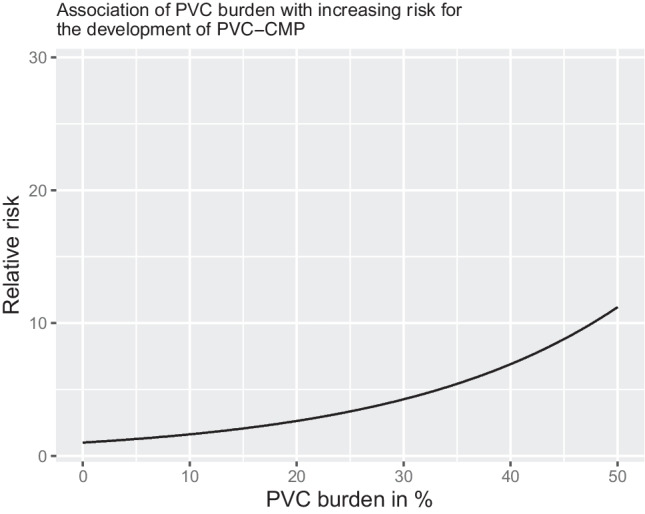
Dose–response plot of PVC burden and association with PVC-CMP. Based on 7 studies reporting PVC burden with a cutoff, a dose–response analysis was conducted. The black line represents the predicted increase in PVC-CMP risk associated with an increase in PVC burden in %. The gray ribbon represents the confidence interval of the prediction

In the dose–response analysis encompassing 7 studies reporting PVC burden at different cutoffs, there was a highly significant association between increase in PVC burden and an exponential increase in risk for PVC-CM (at 10% PVC burden, beta-coefficient 1.54 [1.3, 1.8], at 20% PVC burden beta-coefficient 1.5 [1.7, 3.6], at 30% PVC burden beta-coefficient 4 [2.3, 7], Fig. 10). A univariate Cochran *Q* test for residual heterogeneity was highly significant, with an *I*^2^ statistic of 89.7%.

### Modification of the risk associated with PVC burden through meta-regression

When assessing the risk modification associated with the publication year or with study quality, older studies and studies with higher quality were associated with a non-significant trend in increased risk for the development of PVC-CM with a growing PVC burden.

The PVC-CM risk associated with PVC burden decreased of 0.28% (− 0.28%, 95% CI [− 1.02%, 0.46%], *P* = 0.462, Supplemental Fig. 2) with each increase in publication year, meaning that studies published in 2020 displayed a non-significant 2.8% lower risk association of PVC-CM with PVC burden as compared with the studies published in 2010.

Inversely, the PVC-CM risk associated with PVC burden increased of 0.09% (95% CI [− 0.13%, 0.31%], *P* = 0.413, Supplemental Fig. 3) with each increase in quality point of the summed QUIPS tool, meaning that studies with a low risk of bias (in mean 45 points in the summed QUIPS tool) presented a 2.7% higher risk association of PVC-CM with PVC burden as compared with the studies with high risk of bias (in mean 15 points in the summed QUIPS tool).

### Publication bias

**Fig. 11:**
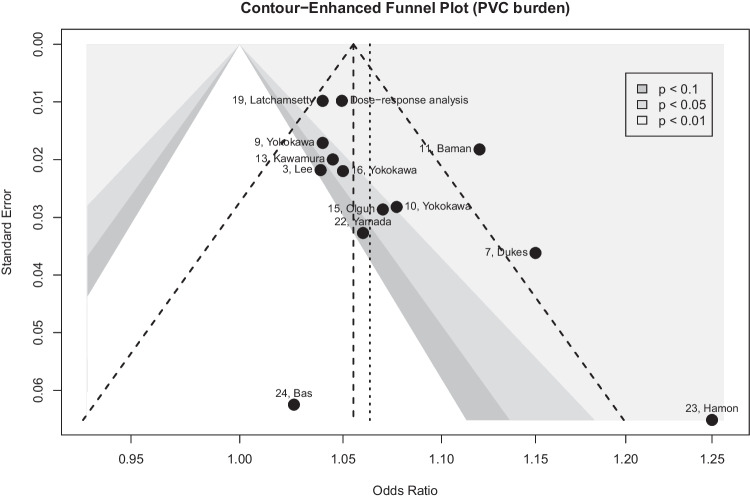
Assessment of publication bias using a contour-enhanced funnel plot. The contour-enhanced funnel plot represents the different studies reporting estimated for the association between PVC burden (continuous increase in %) and assess the risk for publication bias. The 7 studies reporting a cutoff of PVC burden were summarized beforehand as the “dose–response analysis.” The dotted line represents the overall estimate using all available studies and the dashed line represents a classical funnel plot with the expected distribution of the studies if no publication bias is present. The contour-enhanced funnel plot is centered at 0 (i.e., the value under the null hypothesis of no relationship) and various levels of statistical significance are indicated by the shaded region. The white region corresponds to non-significant *P* values. Highly significant *P* values appear in the light gray region

On funnel plot analysis of PVC burden, study distribution was mildly asymmetric (Fig. 11) but the Egger test did not suggest any publication bias (*P* = 0.07).

### Quality assessment

**Fig. 12:**
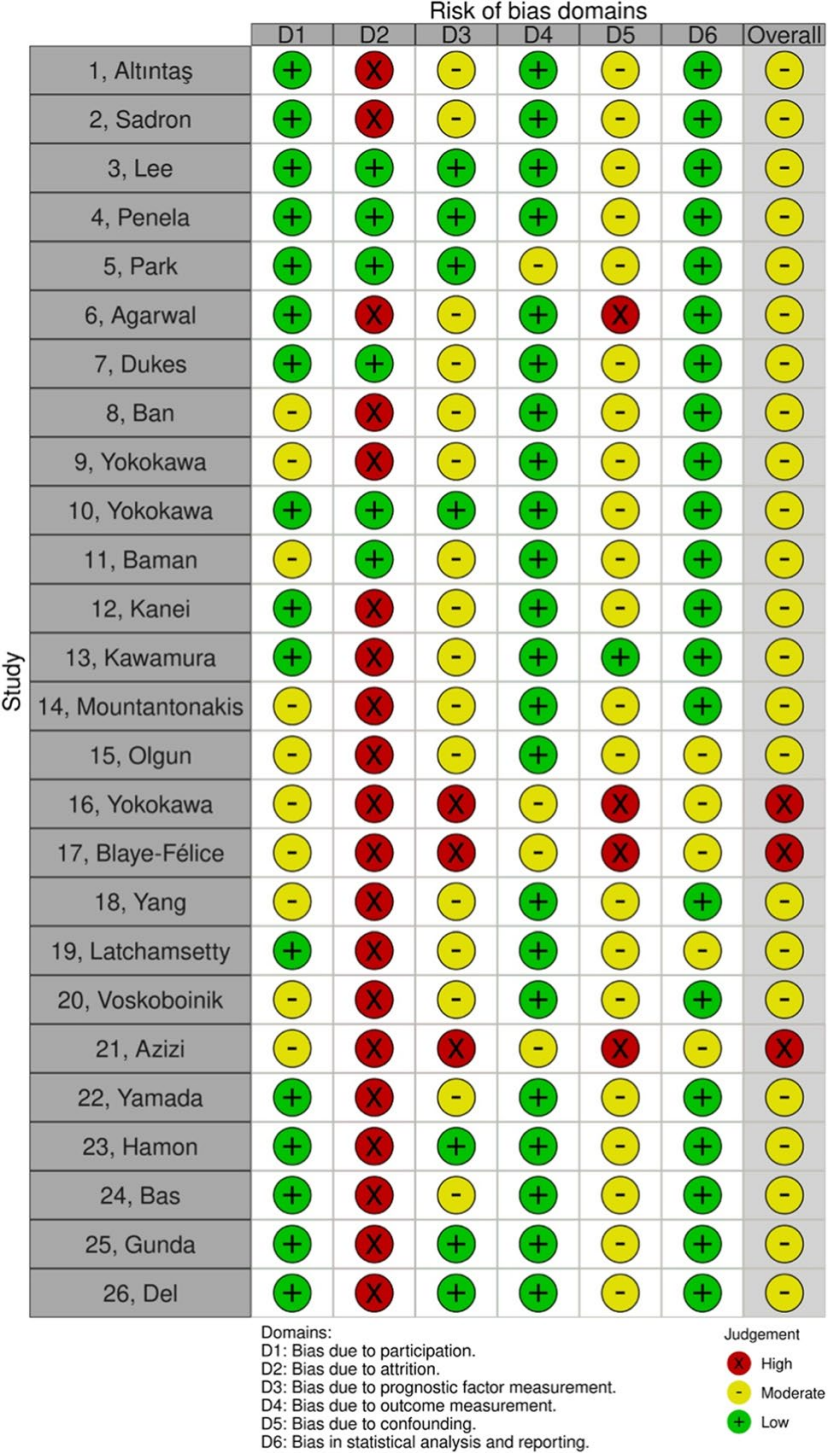
Assessment of study quality. Evaluation of study quality according to the QUIPS tool. Five domains of bias (participation, attrition, prognostic factor measurement, outcome measurement, confounding and statistical analysis and reporting) are represented with the associated risk of bias (high in red, moderate in yellow, and low in green). The overall column represents the mean risk of bias from the 6 domains

As presented in Fig. 12, all of the studies presented with at least a moderate risk of bias. The uncontrolled risk of confounding appeared as the most problematic throughout all recorded studies.

## Discussion

This systematic review and meta-analysis investigated 26 studies to investigate risk factors associated with the development of PVC-CM. We report *four* major findings. *First*, despite screening abstracts published over 30 years of scientific research, only few studies presented a multivariable assessment of risk factors potentially associated with PVC-CM and the quality of the research currently does not allow for definitive conclusion. *Second*, although many candidate risk factors were proposed by the analyzed studies, only 13 risk factors (age, PVC burden, PVC origin from epicardial, outflow tract or LV, interpolation, non-sustained VTs, presence of symptoms, coupling interval, PVC morphology and duration, QRS duration, and sex) were reported often enough with appropriate statistics to allow for a quantitative summary. Many other predictors remain possible candidates for the risk stratification of PVC-CM development. *Third*, age, non-sustained VTs, LV and epicardial origin, interpolation, PVC duration, and PVC burden were all associated with an increased risk for PVC-CM, whereas the presence of symptoms significantly reduced the risk. *Fourth*, there was a clear association between increasing PVC burden and increasing PVC-CM risk. In the dose–response analysis encompassing 7 studies reporting PVC burden at different cutoffs, there was a highly significant association between increase in PVC burden and an increasing risk for PVC-CM. Specifically, per % increase in PVC burden, there was an exponential increase in the absolute risk of PVC-CM. This association was not significantly impacted by the study publication year, suggesting that despite improvements in heart failure treatments and prevention over years, the burden remains an important predictor of PVC-CM development.

To the best of our knowledge, this is the first systematic review and meta-analysis comprehensively assessing the risk factors for the development of PVC-induced cardiomyopathy. The optimal approach to frequent PVCs (> 10% burden) without LV dysfunction, symptoms, or idiopathic ventricular fibrillation is unclear, but patients should probably be monitored every 6–12 months with echocardiography and PVC burden assessment [47]. Therefore, until PVC-induced cardiomyopathy can be predicted, these results help to focus on patients at the highest risk of developing PVC-CM. The role of early rhythm control with catheter ablation or AAD of frequent PVCs without LV dysfunction and symptoms, but risk factors, needs to be defined.

Several studies have confirmed a correlation between a higher PVC burden and development of cardiomyopathy, although no precise burden of PVCs consistently predicts the development of a cardiomyopathy. In this meta-analysis, we found a highly significant association between an increase in PVC burden and increasing risk for PVC-CM.

### Limitations

This systematic review and meta-analysis has several limitations. First, the quantitative summary of risk factors we are presenting summarizes different measures of risks (odds and hazard ratios) together. While this has been conducted in previous research and is acknowledged by recent guidelines as a possible necessary simplification [14], this might have biased absolute risk estimated. Second, most of the articles had different definitions for the risk factors. As such, only 15 of the 26 analyzed studies (57%) provided a definition for PVC-CM and only 9 of the 26 (34.6%) assessed the evolution of EF into the model. The latest literature on PVC-CM [2, 48, 49] recommends assessing the temporal course of worsening or recovery of EF over time. Thus, about three fourth of the studies we investigated did not define their main endpoint with enough precision. At the same time, none of the three included studies provided a standardized definition for non-sustained tachycardia, limiting the credibility of the result.

Third, as several studies did not thoroughly assess other underlying heart failure etiologies in their patients collectively, our estimates may have been occasionally confounded by other causes of heart failure. Fourth, as most of the studies providing a PVC burden cutoff only provided two categories, we had to assume a linear trend between PVC exposure and the associated increase in risk (thereby leading to an exponentially growing risk after back-transformation of the log-odds). With more detailed data, quadratic estimations could lead to more accurate dose–response relationship modelling.

## Conclusion

In this meta-analysis, the most consistent risk factors for PVC-CM were age, non-sustained VTs, LV and epicardial origin, interpolation, PVC duration, and PVC burden, while the presence of symptoms significantly reduced the risk. These findings help tailor stringent follow-up to patients presenting with frequent PVCs and normal LV function.

## Supplementary information

Below is the link to the electronic supplementary material.

Supplementary file1 (DOCX 861 KB)
