# Supplementary material for: Risk factors for the development of premature ventricular complex-induced cardiomyopathy: a systematic review and meta-analysis
Source: J Interv Card Electrophysiol. 2022 Nov 21;66(5):1145–63. doi: 10.1007/s10840-022-01421-8 (PMC10333144; doi:10.1007/s10840-022-01421-8)
Supplement: Supplementary file 1 — Supplementary file1 (DOCX 861 KB) [file 10840_2022_1421_MOESM1_ESM.docx]

# Supplemental appendix

[Supplemental appendix 1](#_Toc117425295)

[Literature search 3](#_Toc117425296)

[General search strategy 3](#_Toc117425297)

[Pubmed and MEDLINE search strategy 3](#_Toc117425298)

[Embase search strategy 4](#_Toc117425299)

[Supplemental methods 7](#_Toc117425300)

[Study selection and data preparation 7](#_Toc117425301)

[Data extraction 8](#_Toc117425302)

[Primary endpoint 8](#_Toc117425303)

[Analysis of risk factors 8](#_Toc117425304)

[Statistical methods 9](#_Toc117425305)

[Definition of Risk Factors 10](#_Toc117425306)

[Supplemental table 1 - PRISMA reporting guidelines 12](#_Toc117425307)

[Supplemental table 2 – Review form for the initial literature review 15](#_Toc117425308)

[Supplemental table 3 – detailed study characteristics 16](#_Toc117425309)

[Supplemental table 4 – Diagnostics conducted 26](#_Toc117425310)

[Supplemental table 5 – Details of the conducted diagnostics in each study 28](#_Toc117425311)

[Supplemental table 6 – Assessment of risk factors 31](#_Toc117425312)

[Supplemental Figure 1 1-4 – Meta-analytic summaries for further, less common risk factors 35](#_Toc117425313)

[Supplemental figure 2 – Impact of the publication year on the risk of PVC-CM associated with PVC burden. 37](#_Toc117425314)

[Supplemental figure 3 - Impact of the study quality on the risk of PVC-CM associated with PVC burden. 38](#_Toc117425315)

[References 39](#_Toc117425316)

## Literature search

### General search strategy

To ensure completeness of the search we used both words or truncated words and MESH terms. No limitations of languages were applied. References list of each article was reviewed for potential missed studies.

### Pubmed and MEDLINE search strategy

(((heart ventricle extrasystole[Title/Abstract]) OR (cardiac ventricle ectopic beat[Title/Abstract]) OR (cardiac ventricle extrasystole[Title/Abstract]) OR (cardiac ventricular ectopic beat[Title/Abstract]) OR (cardiac ventricular extrasystole[Title/Abstract]) OR (ectopic ventricular beat[Title/Abstract]) OR (electrocardiography, interpolated ventricular beat[Title/Abstract]) OR (extrasystole, heart ventricle[Title/Abstract]) OR (heart ventricle ectopic beat[Title/Abstract]) OR (heart ventricle escape beat[Title/Abstract]) OR (heart ventricle extra systole[Title/Abstract]) OR (heart ventricle extrasystole[Title/Abstract]) OR (heart ventricle premature beat[Title/Abstract]) OR (heart ventricle premature systole[Title/Abstract]) OR (heart ventricular ectopic beat[Title/Abstract]) OR (heart ventricular extra systole[Title/Abstract]) OR (heart ventricular extrasystole[Title/Abstract]) OR (heart ventricular premature beat[Title/Abstract]) OR (interpolated heart ventricle beat[Title/Abstract]) OR (interpolated heart ventricular beat[Title/Abstract]) OR (premature heart ventricle contraction[Title/Abstract]) OR (premature ventricle contraction[Title/Abstract]) OR (premature ventricular beat[Title/Abstract]) OR (premature ventricular contraction[Title/Abstract]) OR (ventricle ectopic beat, heart[Title/Abstract]) OR (ventricle extrasystole[Title/Abstract]) OR (ventricular ectopic beat[Title/Abstract]) OR (ventricular extra systole[Title/Abstract]) OR (ventricular extrasystole[Title/Abstract]) OR (ventricular extrasystolia[Title/Abstract]) OR (ventricular parasystole[Title/Abstract]) OR (ventricular premature beat[Title/Abstract]) OR (ventricular premature complex[Title/Abstract]) OR (ventricular premature complexes[Title/Abstract]) OR (ventricular premature depolarisation[Title/Abstract]) OR (ventricular premature depolarization[Title/Abstract]) OR (‘ventricular premature depolarization’[Title/Abstract]) OR (premature ventricular beat[MeSH Terms]) OR (premature ventricular complex[MeSH Terms]) OR (Ventricular Premature Complexes[MeSH Terms])) AND ((Risk Factors[MeSH Terms]) OR (Risk assessment[MeSH Terms]) OR (protective factors[MeSH Terms]) OR (precipitating factors[MeSH Terms]) OR (Multivariate Analysis[MeSH Terms]) OR (Correlation of Data[MeSH Terms]) OR (Risk Factors[MeSH Terms]) OR (Risk Assessment[MeSH Terms]) OR (Risk Adjustment[MeSH Terms]) OR (Logistic Models[MeSH Terms]) OR (Linear Models[MeSH Terms]) OR (Regression Analysis[MeSH Terms]) OR (Protective Factors [MeSH Terms]) OR (Time Factors[MeSH Terms]) OR (Risk Factors[Title/Abstract]) OR (Risk assessment[Title/Abstract]) OR (protective factors[Title/Abstract]) OR (precipitating factors[Title/Abstract]) OR (Multivariate Analysis[Title/Abstract]) OR (Correlation of Data[Title/Abstract]) OR (Risk Factors[Title/Abstract]) OR (Risk Assessment[Title/Abstract]) OR (Risk Adjustment[Title/Abstract]) OR (Logistic Models[Title/Abstract]) OR (Linear Models[Title/Abstract]) OR (Regression Analysis[Title/Abstract]) OR (Protective Factors [Title/Abstract]) OR (Time Factors[Title/Abstract]) OR (predictors[Title/Abstract]) OR (multivariate[Title/Abstract]) OR (multivariable[Title/Abstract]) OR (risk factor[Title/Abstract]) OR (relative risk[Title/Abstract]) OR (risk factor[Title/Abstract]) OR (risk factors[Title/Abstract]) OR (risk assessment[Title/Abstract]) OR (assessment, safety[Title/Abstract]) OR (risk adjustment[Title/Abstract]) OR (risk analysis[Title/Abstract]) OR (risk assessment[Title/Abstract]) OR (risk evaluation[Title/Abstract]) OR (safety assessment[Title/Abstract]) OR (multivariate analysis[Title/Abstract]) OR (analysis, multivariate[Title/Abstract]) OR (multivariate analysis[Title/Abstract]) OR (multivariate statistical data[Title/Abstract]) OR (multivariate statistics[Title/Abstract]) OR (statistical data, multivariate[Title/Abstract]) OR (multivariate logistic regression analysis[Title/Abstract]) OR (logistic regression analysis, multivariate[Title/Abstract]) OR (multiple logistic regression[Title/Abstract]) OR (multivariate logistic regression analysis[Title/Abstract]) OR (regression analysis, multivariate logistic[Title/Abstract]) OR (protection[Title/Abstract]) OR (protection[Title/Abstract]) OR (protective factors[Title/Abstract]) OR (causality[Title/Abstract]) OR (causality[Title/Abstract]) OR (correlational study[Title/Abstract]) OR (correlation studies[Title/Abstract]) OR (correlation study[Title/Abstract]) OR (correlational studies[Title/Abstract]) OR (correlational study[Title/Abstract]) OR (correlation analysis[Title/Abstract]) OR (canonical correlation analysis[Title/Abstract]) OR (correlation analysis[Title/Abstract]) OR (correlational analysis[Title/Abstract]) OR (correlation coefficient[Title/Abstract]) OR (kendalls tau[Title/Abstract]) OR (kendalls tau correlation coefficient[Title/Abstract]) OR (pearsons correlation coefficient[Title/Abstract]) OR (spearmans rank correlation coefficient[Title/Abstract]) OR (correlation coefficient[Title/Abstract]) OR (intraclass correlation coefficient[Title/Abstract]) OR (time factor[Title/Abstract]) OR (time factor[Title/Abstract]) OR (time factors[Title/Abstract]))) AND ("Cardiac Output, Low"[Mesh] OR "Cardiomyopathy, Dilated"[Mesh] OR "Heart Failure"[Mesh:NoExp] OR "Edema, Cardiac"[Mesh] OR "Heart Failure, Systolic"[Mesh] OR "Ventricular Dysfunction"[Mesh] OR 'acute heart failure'[Title/Abstract] OR 'cardiopulmonary insufficiency'[Title/Abstract] OR 'systolic heart failure'[Title/Abstract] OR 'heart edema'[Title/Abstract] OR 'forward heart failure'[Title/Abstract] OR 'heart left ventricle failure'[Title/Abstract] OR 'heart left ventricle overload'[Title/Abstract] OR 'systolic dysfunction'[Title/Abstract] OR 'nonischemic cardiomyopathy'[Title/Abstract] OR 'cardiomyopathy'[Title/Abstract] OR 'heart dilatation'[Title/Abstract] OR 'heart failure'[Title/Abstract] OR ‘left ventricular dysfunction’[Title/Abstract] OR ‘cardiac decompensation’[Title/Abstract] OR ‘cardiac insufficiency’[Title/Abstract] OR ‘cardiac failure’[Title/Abstract] OR ‘cardiac incompentence’[Title/Abstract] OR ‘cardial decompensation’[Title/Abstract] OR ‘cardial insufficiency’[Title/Abstract] OR ‘cardial failure’[Title/Abstract] OR ‘cardial incompentence’[Title/Abstract] OR ‘myocardial decompensation’[Title/Abstract] OR ‘myocardial insufficiency’[Title/Abstract] OR ‘myocardial failure’[Title/Abstract] OR ‘myocardial incompetence’[Title/Abstract]) NOT (animals [mh] NOT humans [mh])NOT ((Adolescent[mesh] OR child[mesh] OR infant[mesh]) NOT adult[mesh])

### Embase search strategy

('ventricular premature contraction'/exp OR 'heart ventricle extrasystole'/exp OR 'cardiac ventricle ectopic beat' OR 'cardiac ventricle extrasystole' OR 'cardiac ventricular ectopic beat' OR 'cardiac ventricular extrasystole' OR 'ectopic ventricular beat' OR 'electrocardiography, interpolated ventricular beat' OR 'extrasystole, heart ventricle' OR 'heart ventricle ectopic beat' OR 'heart ventricle escape beat' OR 'heart ventricle extra systole' OR 'heart ventricle extrasystole' OR 'heart ventricle premature beat' OR 'heart ventricle premature systole' OR 'heart ventricular ectopic beat' OR 'heart ventricular extra systole' OR 'heart ventricular extrasystole' OR 'heart ventricular premature beat' OR 'interpolated heart ventricle beat' OR 'interpolated heart ventricular beat' OR 'premature heart ventricle contraction' OR 'premature ventricle contraction' OR 'premature ventricular beat' OR 'premature ventricular contraction' OR 'ventricle ectopic beat, heart' OR 'ventricle extrasystole' OR 'ventricular ectopic beat' OR 'ventricular extra systole' OR 'ventricular extrasystole' OR 'ventricular extrasystolia' OR 'ventricular parasystole' OR 'ventricular premature beat' OR 'ventricular premature complex' OR 'ventricular premature complexes' OR 'ventricular premature depolarisation' OR 'ventricular premature depolarization' OR (heart ventricle extrasystole:ab,ti ) OR (cardiac ventricle ectopic beat:ab,ti ) OR (cardiac ventricle extrasystole:ab,ti ) OR (cardiac ventricular ectopic beat:ab,ti ) OR (cardiac ventricular extrasystole:ab,ti ) OR (ectopic ventricular beat:ab,ti ) OR (electrocardiography, interpolated ventricular beat:ab,ti ) OR (extrasystole, heart ventricle:ab,ti ) OR (heart ventricle ectopic beat:ab,ti ) OR (heart ventricle escape beat:ab,ti ) OR (heart ventricle extra systole:ab,ti ) OR (heart ventricle extrasystole:ab,ti ) OR (heart ventricle premature beat:ab,ti ) OR (heart ventricle premature systole:ab,ti ) OR (heart ventricular ectopic beat:ab,ti ) OR (heart ventricular extra systole:ab,ti ) OR (heart ventricular extrasystole:ab,ti ) OR (heart ventricular premature beat:ab,ti ) OR (interpolated heart ventricle beat:ab,ti ) OR (interpolated heart ventricular beat:ab,ti ) OR (premature heart ventricle contraction:ab,ti ) OR (premature ventricle contraction:ab,ti ) OR (premature ventricular beat:ab,ti ) OR (premature ventricular contraction:ab,ti ) OR (ventricle ectopic beat, heart:ab,ti ) OR (ventricle extrasystole:ab,ti ) OR (ventricular ectopic beat:ab,ti ) OR (ventricular extra systole:ab,ti ) OR (ventricular extrasystole:ab,ti ) OR (ventricular extrasystolia:ab,ti ) OR (ventricular parasystole:ab,ti ) OR (ventricular premature beat:ab,ti ) OR (ventricular premature complex:ab,ti ) OR (ventricular premature complexes:ab,ti ) OR (ventricular premature depolarisation:ab,ti ) OR (ventricular premature depolarization:ab,ti ) OR ‘ventricular premature depolarization’:ab,ti)

AND

('risk factor'/exp OR 'relative risk' OR 'risk factor' OR 'risk factors' OR 'risk assessment'/exp OR 'risk adjustment' OR 'risk analysis' OR 'risk assessment' OR 'risk evaluation' OR 'multivariate analysis'/exp OR 'analysis, multivariate' OR 'multivariate analysis' OR 'multivariate statistical data' OR 'multivariate statistics' OR 'statistical data, multivariate' OR 'multivariate logistic regression analysis'/exp OR 'logistic regression analysis, multivariate' OR 'multiple logistic regression' OR 'multivariate logistic regression analysis' OR 'regression analysis, multivariate logistic' OR 'protection'/exp OR 'protection' OR 'protective factors' OR 'causality'/exp OR 'causality' OR 'correlational study'/exp OR 'correlation studies' OR 'correlation study' OR 'correlational studies' OR 'correlational study' OR 'correlation analysis'/exp OR 'correlation analysis' OR 'correlational analysis' OR 'correlation coefficient'/exp OR (Risk Factors:ab,ti ) OR (Risk assessment:ab,ti ) OR (protective factors:ab,ti ) OR (precipitating factors:ab,ti ) OR (Multivariate Analysis:ab,ti ) OR (Risk Factors:ab,ti ) OR (Risk Assessment:ab,ti ) OR (Risk Adjustment:ab,ti ) OR (Logistic Models:ab,ti ) OR (Linear Models:ab,ti ) OR (Regression Analysis:ab,ti ) OR (Protective Factors:ab,ti ) OR (predictors:ab,ti ) OR (multivariate:ab,ti ) OR (multivariable:ab,ti ) OR (risk factor:ab,ti ) OR (relative risk:ab,ti ) OR (risk factor:ab,ti ) OR (risk factors:ab,ti ) OR (risk assessment:ab,ti ) OR (risk adjustment:ab,ti ) OR (risk analysis:ab,ti ) OR (multivariate analysis:ab,ti ) OR (analysis, multivariate:ab,ti ) OR (multivariate analysis:ab,ti ) OR (multivariate statistical data:ab,ti ) OR (multivariate statistics:ab,ti ) OR (statistical data, multivariate:ab,ti ) OR (multivariate logistic regression analysis:ab,ti ) OR (logistic regression analysis, multivariate:ab,ti ) OR (multiple logistic regression:ab,ti ) OR (multivariate logistic regression analysis:ab,ti ) OR (regression analysis, multivariate logistic:ab,ti ) OR (protective factors:ab,ti ))

AND

'heart failure'/exp OR 'backward failure, heart' OR 'cardiac backward failure' OR 'cardiac decompensation' OR 'cardiac failure' OR 'cardiac incompetence' OR 'cardiac insufficiency' OR 'cardiac stand still' OR 'cardial decompensation' OR 'cardial insufficiency' OR 'chronic heart failure' OR 'chronic heart insufficiency' OR 'decompensatio cordis' OR 'decompensation, heart' OR 'heart backward failure' OR 'heart decompensation' OR 'heart failure' OR 'heart incompetence' OR 'heart insufficiency' OR 'insufficientia cardis' OR 'myocardial failure' OR 'myocardial insufficiency' OR 'cardiopulmonary failure'/exp OR 'cardiomyopathy'/exp OR 'cardiomyopathies' OR 'cardiomyopathy' OR 'heart myopathy' OR 'myocardiopathy' OR 'primary myocardial disease' OR 'heart left ventricle failure'/exp OR 'heart left ventricle dysfunction' OR 'heart left ventricle failure' OR 'heart left ventricle insufficiency' OR 'left cardiac failure' OR 'left heart failure' OR 'left heart insufficiency' OR 'left heart ventricle failure' OR 'left heart ventricle insufficiency' OR 'left heart ventricular failure' OR 'left ventricle failure' OR 'left ventricular dysfunction' OR 'left ventricular failure' OR 'left ventricular heart failure' OR 'acute heart failure'/exp OR 'cardiopulmonary insufficiency'/exp OR 'systolic heart failure'/exp OR 'heart edema'/exp OR 'forward heart failure'/exp OR 'heart left ventricle failure'/exp OR 'heart left ventricle overload'/exp OR 'systolic dysfunction'/exp OR 'nonischemic cardiomyopathy'/exp OR 'cardiomyopathy'/de OR 'heart dilatation'/exp OR 'heart failure'/de OR 'acute heart failure':ab,ti OR 'cardiopulmonary insufficiency':ab,ti OR 'systolic heart failure':ab,ti OR 'heart edema':ab,ti OR 'forward heart failure':ab,ti OR 'heart left ventricle failure':ab,ti OR 'heart left ventricle overload':ab,ti OR 'systolic dysfunction':ab,ti OR 'nonischemic cardiomyopathy':ab,ti OR 'cardiomyopathy':ab,ti OR 'heart dilatation':ab,ti OR 'heart failure':ab,ti OR ‘left ventricular dysfunction’:ab,ti OR ‘cardiac decompensation’:ab,ti OR ‘cardiac insufficiency’:ab,ti OR ‘cardiac failure’:ab,ti OR ‘cardiac incompentence’:ab,ti OR ‘cardial decompensation’:ab,ti OR ‘cardial insufficiency’:ab,ti OR ‘cardial failure’:ab,ti OR ‘cardial incompentence’:ab,ti OR ‘myocardial decompensation’:ab,ti OR ‘myocardial insufficiency’:ab,ti OR ‘myocardial failure’:ab,ti OR ‘myocardial incompentence’:ab,ti

NOT ('animal'/exp OR 'nonhuman'/exp NOT ('animal'/exp OR 'nonhuman'/exp AND 'human'/exp))

NOT (('infant'/exp OR 'child'/exp OR 'adolescent'/exp) NOT 'adult'/exp)

## Supplemental methods

### Study selection and data preparation

The search design was conducted with the assistance of a research librarian. All initially identified studies were exported to the Mendeley Citation Software for duplicate removal. The first step of the screening consisted of an independent review by two members of the study team (JM, LM) of the study title and abstract. A structured questionnaire (Supplemental table 2) was followed for this first selection. A third member of the team (JdFdL) resolved disagreements by discussion. Review of full text and final selection of studies was performed by three independent members of the study team (JdFdL, JM, LM). Disagreements were again resolved by discussion.

We excluded reviews, other meta-analyses, comments or editorials.

While premature ventricular complex induced cardiomyopathy (PVC-CM) can only be definitively diagnosed after exclusion of other causes of heart failure^1–3^, we suspected that the large amount of investigations necessary for this purpose would lead to the exclusion of several studies. Therefore, we did not predefine any necessary comprehensive diagnostic work-up to exclude other causes of heart failure (ischemic or valvular).

We did not include studies reporting summary statistics from machine learning processes, Area Under the Receiving Operating Curve (AUC), sensitivity, specificity or that derived risk prediction tools such as risk charts.

When models were assessed on different populations (Patients with confirmed PVC-CM, control patients without heart failure or with heart failure from other causes for instance), all reported information was considered. However, we excluded studies on special populations (patients with exercise-induced PVC-CM, patients with arrhythmogenic right ventricular cardiomyopathy (ARVC), pregnant women, patients with rare genetic mutations). Patients with specific baseline characteristics (of a certain adult age range, with comorbidities such as hypertension or chronic obstructive pulmonary lung disease (COPD), of a certain race) were not considered special populations and these studies were included.

When risk factors were assessed in their univariable relationship to the outcome or incorporated in multivariable models, all provided estimates were extracted. We predefined that if a model was presented for both a derivation and validation cohort, only data stemming from the validation cohort would be assessed. However, only one study^4^ among the finally selected studies presented with a derivation-validation design and the validation was assessed using an AUC, so that we recorded derivation data for this study as well.

In order not to initially limit selected studies, we did not predefine a minimal set of comorbidities for which the multivariable models should correct^5^.

Studies were excluded if there was no evaluation to exclude causes of heart failure other than PVCs, non-original research publications, specific populations such as pregnant women or patients with exercise-induced PVCs.

### Data extraction

Two members of the study team (LM or JM and JdFdL) reviewed each selected study for data extraction in a dedicated RedCap database hosted at the University Hospital of Basel. Disagreements were solved by discussion. Extracted data included study and patients populations characteristics, details of the outcome definitions, interventions and diagnostics, modelled risk factors and statistical summaries.

### Primary endpoint

The primary outcome of this meta-analysis was the presence of PVC-CM, which we pre-defined either as the development , presence or recovery from heart failure with reduced ejection fraction (HFrEF) in patients with CMP in whom no other cause of heart failure was evident. Accordingly, for our primary analysis we did not differentiate between studies assessing the presence of HFrEF, the worsening of EF or a recovery of EF as all of these processes are part of the diagnosis of PVC-CM^6–8^. A pre-defined minimum EF worsening or recovery was not specified, as there is currently no absolute consensus in modern literature^6–8^. To acknowledge this lack of standardization in the definition of PVC-CM we also included studies reporting a continuous change in EF believed to be due to PVCs to allow for at least qualitative comparison.

Patients with PVC and heart failure with preserved ejection fraction were not considered as potentially presenting with PVC-CM^6–8^.

### Analysis of risk factors

Each risk factor described in the selected studies were first recorded using their name, and when available exact definition. As previously stated, we predefined that a quantitative meta-analysis would be conducted on each factor that occurred ≥3 times with a similar definition throughout analyzed studies. Other risk factors were considered as “candidate” risk factors and presented qualitatively.

When risk factors occurred in ≥ 3 studies, the exact estimates and measures of uncertainty associated with the risk factors were recorded.

Before proceeding with quantitative analysis, we ensured that the tested and reference groups were always comparable. For instance, if studies reported the absence of symptoms as a risk factor, the quantitative risk estimates were inverted in order to be quantitatively combined with studies reporting the presence of symptoms as risk factor. Similarly, when female sex was tested as a risk in certain studies, the risk estimates were inverted in order to be combined with studies testing male sex as a risk factor.

When risk factors were presented against different but compatible reference categories, these risk factors were combined for quantitative analysis. For instance, when left ventricular PVC origin was compared with “other PVC origins” or “Right ventricular PVC origins”, we assumed that both reference categories represented the same entity.

### Statistical methods

Many observational and epidemiologic studies of associations between an exposure and a disease report a dose-response relation in terms of relative risk for groups' exposure levels and the cut-offs between these different groups may be chosen arbitrarily or depending on the underlying risk distribution in the observed population. This leads to great variability between studies. In an attempt to homogenize these observations, Greenland and Longnecker^9^ developed a specific type of meta-analysis to reconstruct and combine study-specific curves from summarized dose-response data. Therefore, when continuous risk factors were presented using cut-offs, the exposure per group (above and below the respective cut-off) was derived as recommended in previous dose-exposure meta-analyses and corresponding guidelines^10–13^: If a range of exposure was reported for a certain continuous risk factor, we estimated the approximate median using the midpoints of the lower and upper bounds. For risk factor exposure reported with an open-ended highest exposure category, we assumed that the difference from the lowest range of this category and its median was equivalent to the difference between the lowest range of the closest adjacent category and it’s median. For example, Park et al.^14^ reported the exposure to PVC burden as ≥ 26% per day. The estimated median PVC exposure calculated for these categories for pooled analysis were 13% and 39% per day respectively. Using the “dosresmeta”^15^ package available in the R Programming language, we computed the pooled dose-response association using a random effect model through restricted maximum likelihood. For each study, the number of patients in the reference group (OR 1) and in the comparison groups with their respective ORs (with confidence intervals) and corresponding cut-offs were fed into the model. A two-stage analysis was conducted to estimate a dose-response association first in each study separately and then in all studies reporting the same risk factors.

To estimate the between-study variance, the Tau estimator was calculated according to the DerSimonian-Laird estimator.^16,17^

The DerSimonian and Laird method is a standard method to conduct a random-effect meta-analysis, a meta-analysis assuming that the observed estimates of the impact of a risk factor can vary across studies not only because of real differences in the impact of this risk factor but also because of sample variability (chance). To undertake a random-effects meta-analysis, the standard errors of the study-specific estimates are adjusted to incorporate a measure of the extent of variation, or heterogeneity, among the intervention effects observed in different studies (this variation is often referred to as tau-squared). The amount of variation, and hence the adjustment, can be estimated from the intervention effects and standard errors of the studies included in the meta-analysis. In order to conduct the present random-effect meta-analysis we used the package "metagen" in the "R statistical language"

The estimates obtained in the dose-response analysis were used as one combined estimate in the random effect models.

We recorded the number of patients and outcomes per groups as well as estimates with 95% confidence intervals and p-values, when available. When part of these quantitative measures were missing, we attempted to derive them from available data. For instance, the number of events per group was derived using OR, total number of patients per group and total number of outcomes. 95% CI were derived from estimates, group sizes and p-values when missing and p-values were computed using 95%-CI, estimates and group sizes as described in previous literature^18^.

We recorded both estimates stemming from univariable and multivariable analysis for each risk factor but used only multivariable estimates when available.

Given the low number of studies available for quantitative summary of most risk factors, heterogeneity was investigated for PVC burden only for study quality and year of publication using meta-regression, as we hypothesized this variable would be available for all studies and could play a possible role in results divergence.

It has been shown and discussed^19^ that a meta-analysis of odds ratios is equivalent to a meta-analysis of effect size when there is an underlying continuous distribution. Thereby using an estimation of the logarithmized ratios (odds or hazard) allows assuming a common underlying log-Normal distribution. As conducted in previous meta-analyses^20,21^ this is how we proceeded using the “MetaGen” package in the R Statistical language.

### Definition of Risk Factors

1. Epicardial origin of the PVC

For epicardial origin, all studies estimated the PVC origin through anatomical mapping and confirmed it through successful ablation of the PVC at the estimated site^22–26^ . None of the studies described the way the differentiation between the epicardial to the mid-myocardial origin of the PVCs was made. The proportion of patients having endocardial/epicardial substrate in the included studies were: 6% and 15%^22^, 83% and 17%^23^, 85% and 15%^24^, 89% and 11%^25^ and 77% and 23%^26^. Surface ECG signals were not used to distinguish PVC locations in the studies investigating PVC origin with one exception, where an ECG algorithm was presented. The patients were nonetheless diagnosed using activation mapping.

1. Interpolated PVCs

In the first study^27^ interpolated PVCs were defined as PVC complexes occurring between two sinus beats without presenting any compensatory pause, as first discribed by Katz et.al.^28^. The two other studies^26,29^ referred to previous research accepting the same definition.

The mean interpolation burden was 21 ± 30%^27^ and 28.2 ± 10.2^26^ while the third study did not provide a mean interpolated PVC burden^29^.

One study^26^ defined it as the presence of interpolation in more than 50% of the PVCs during the first 20 minutes of the electrophysiology study. The two remaining studies did not provide any cut-off value^27,29^

1. Left ventricular PVC origin

The left ventricular PVC origin was estimated in three studies^22,24,26^ invasively during EP study and was defined as the ablation site which led to the termination of the arrhythmia. The origin was mapped by using conventional or tridimensional system-based activation mapping (earliest endocardial activation) together with pace mapping. Park et.al.^30^ used invasive electrophysiological mapping in a fraction of patients, and surface ECG morphologic criteria in the rest of the patients.

1. Non-sustained Ventricular Tachycardia

Non-sustained Ventricular tachycardia was defined by Ban et.al.^31^ as ≥3 consecutive PVCs with a duration of <30s encompassing at least 1% of the overall PVC burden on 24 h Holter monitoring. Kanei et.al.^32^ defined non-sustained VT by ≥3 consecutive PVCs without providing additional criteria, while the article of Voskoboinik et.al.^33^ did not provide any definition.

1. Symptoms

The study by Yokokawa et.al.^34^ reported the presence of heart failure symptoms. The study by Bas et.al.^29^ included in this category patients with palpitations, lightheadedness and syncope. Latchamsetty et.al.^25^ did not provide any details on the type or spectrum of symptoms.

1. Coupling interval

Kawamura et.al. ^35^ reported the coupling interval dispersion as the difference between the maximum and the minimal coupling intervals. Hamon et.al.^26^ did not provide any further detail on their definition of coupling intervals.

1. Polymorphic PVCs

All studies defined polymorphism as several PVC morphologies seen on the surface ECG^24,25,29^.

1. Outflow Tract PVC origin

All studies diagnosed outflow tract PVCs using intraprocedural mapping and confirmed the location by successful ablation at the mapped location^24,25,36^.

## Supplemental table 1 - PRISMA reporting guidelines

| **Section and Topic** | **Item #** | **Checklist item** | **Location where item is reported** |
| --- | --- | --- | --- |
| **TITLE** | | |  |
| Title | 1 | Identify the report as a systematic review. | Title |
| **ABSTRACT** | | |  |
| Abstract | 2 | See the PRISMA 2020 for Abstracts checklist. | Abstract |
| **INTRODUCTION** | | |  |
| Rationale | 3 | Describe the rationale for the review in the context of existing knowledge. | Page 4 |
| Objectives | 4 | Provide an explicit statement of the objective(s) or question(s) the review addresses. | Page 4 |
| **METHODS** | | |  |
| Eligibility criteria | 5 | Specify the inclusion and exclusion criteria for the review and how studies were grouped for the syntheses. | Page 5-6, Supp |
| Information sources | 6 | Specify all databases, registers, websites, organisations, reference lists and other sources searched or consulted to identify studies. Specify the date when each source was last searched or consulted. | Page 5, Supp |
| Search strategy | 7 | Present the full search strategies for all databases, registers and websites, including any filters and limits used. | Supp |
| Selection process | 8 | Specify the methods used to decide whether a study met the inclusion criteria of the review, including how many reviewers screened each record and each report retrieved, whether they worked independently, and if applicable, details of automation tools used in the process. | Page 5-6, Supp |
| Data collection process | 9 | Specify the methods used to collect data from reports, including how many reviewers collected data from each report, whether they worked independently, any processes for obtaining or confirming data from study investigators, and if applicable, details of automation tools used in the process. | Page 5-6, Supp |
| Data items | 10a | List and define all outcomes for which data were sought. Specify whether all results that were compatible with each outcome domain in each study were sought (e.g. for all measures, time points, analyses), and if not, the methods used to decide which results to collect. | Page 5-7, Supp |
|  | 10b | List and define all other variables for which data were sought (e.g. participant and intervention characteristics, funding sources). Describe any assumptions made about any missing or unclear information. | Page 5-7, Supp |
| Study risk of bias assessment | 11 | Specify the methods used to assess risk of bias in the included studies, including details of the tool(s) used, how many reviewers assessed each study and whether they worked independently, and if applicable, details of automation tools used in the process. | Page 7-9, Supp |
| Effect measures | 12 | Specify for each outcome the effect measure(s) (e.g. risk ratio, mean difference) used in the synthesis or presentation of results. | Page 7-9, Supp |
| Synthesis methods | 13a | Describe the processes used to decide which studies were eligible for each synthesis (e.g. tabulating the study intervention characteristics and comparing against the planned groups for each synthesis (item #5)). | Page 7-9, Supp |
|  | 13b | Describe any methods required to prepare the data for presentation or synthesis, such as handling of missing summary statistics, or data conversions. | Page 7-9, Supp |
|  | 13c | Describe any methods used to tabulate or visually display results of individual studies and syntheses. | Page 7-9, Supp |
|  | 13d | Describe any methods used to synthesize results and provide a rationale for the choice(s). If meta-analysis was performed, describe the model(s), method(s) to identify the presence and extent of statistical heterogeneity, and software package(s) used. | Page 7-9, Supp |
|  | 13e | Describe any methods used to explore possible causes of heterogeneity among study results (e.g. subgroup analysis, meta-regression). | Page 7-9, Supp |
|  | 13f | Describe any sensitivity analyses conducted to assess robustness of the synthesized results. | Page 7-9, Supp |
| Reporting bias assessment | 14 | Describe any methods used to assess risk of bias due to missing results in a synthesis (arising from reporting biases). | Page 7-9, Supp |
| Certainty assessment | 15 | Describe any methods used to assess certainty (or confidence) in the body of evidence for an outcome. | Page 7-9, Supp |
| **RESULTS** | | |  |
| Study selection | 16a | Describe the results of the search and selection process, from the number of records identified in the search to the number of studies included in the review, ideally using a flow diagram. | Page 10 |
|  | 16b | Cite studies that might appear to meet the inclusion criteria, but which were excluded, and explain why they were excluded. | Page 10 |
| Study characteristics | 17 | Cite each included study and present its characteristics. | Page 10-11 |
| Risk of bias in studies | 18 | Present assessments of risk of bias for each included study. | Page 13 |
| Results of individual studies | 19 | For all outcomes, present, for each study: (a) summary statistics for each group (where appropriate) and (b) an effect estimate and its precision (e.g. confidence/credible interval), ideally using structured tables or plots. | Page 10-13 |
| Results of syntheses | 20a | For each synthesis, briefly summarise the characteristics and risk of bias among contributing studies. | Page 10-13 |
|  | 20b | Present results of all statistical syntheses conducted. If meta-analysis was done, present for each the summary estimate and its precision (e.g. confidence/credible interval) and measures of statistical heterogeneity. If comparing groups, describe the direction of the effect. | Page 10-13 |
|  | 20c | Present results of all investigations of possible causes of heterogeneity among study results. | Page 10-13 |
|  | 20d | Present results of all sensitivity analyses conducted to assess the robustness of the synthesized results. | Page 10-13 |
| Reporting biases | 21 | Present assessments of risk of bias due to missing results (arising from reporting biases) for each synthesis assessed. | Page 10-13 |
| Certainty of evidence | 22 | Present assessments of certainty (or confidence) in the body of evidence for each outcome assessed. | Page 10-13 |
| **DISCUSSION** | | |  |
| Discussion | 23a | Provide a general interpretation of the results in the context of other evidence. | Page 14 |
|  | 23b | Discuss any limitations of the evidence included in the review. | Page 14-15 |
|  | 23c | Discuss any limitations of the review processes used. | Page 14-15 |
|  | 23d | Discuss implications of the results for practice, policy, and future research. | Page 14-15 |
| **OTHER INFORMATION** | | |  |
| Registration and protocol | 24a | Provide registration information for the review, including register name and registration number, or state that the review was not registered. | Page 5 |
|  | 24b | Indicate where the review protocol can be accessed, or state that a protocol was not prepared. | Page 5 |
|  | 24c | Describe and explain any amendments to information provided at registration or in the protocol. | - |
| Support | 25 | Describe sources of financial or non-financial support for the review, and the role of the funders or sponsors in the review. | Page 1-2 |
| Competing interests | 26 | Declare any competing interests of review authors. | Page 1-2 |
| Availability of data, code and other materials | 27 | Report which of the following are publicly available and where they can be found: template data collection forms; data extracted from included studies; data used for all analyses; analytic code; any other materials used in the review. | Supp. |

## Supplemental table 2 – Review form for the initial literature review

| **Field Label** | **Field choices** |
| --- | --- |
| Exclusion based on title and abstract screening only | 1, Yes  2, No |
| Is the article a research article on humans? | 1, Yes  2, No |
| Is the article focusing on a highly selected population? | 1, Yes  2, No |
| Is this a review or a meta-analysis ? | 1, Yes  2, No |
| Is this a study with >50 patients? | 1, Yes  2, No |
| Is the topic on premature ventricular complexes and heart failure? | 1, Yes  2, No |
| What kind of risk factors does the study discuss? | 1, no risk factors  2, RF for the development of PVC-induced CM  3, RF for the development of an adverse outcome in PVC-induced CM  4, both (RF for the development of PVC-induced CM and for the development of an adverse outcome) |
| There is at least one multivariable model in the study | 1, Yes  2, No |
| Exclusion after full-text review | 1, no estimates available  2, missing number of patients per groups  3, improper statistics  4, journal discontinued  5, language of the full paper not assessable  6, none of the risk factors of the paper came in our detailed analysis  7, abstract empty and no corresponding paper  8, Study included |

## Supplemental table 3 – detailed study characteristics

| **Nr** | **Main author** | **Title** | **Inclusion criterion** | **Exclusion criterion** | **PVC-CM Definition present?** | **PVC-CM Definition** | **PVC definition present?** | **PVC definition** |
| --- | --- | --- | --- | --- | --- | --- | --- | --- |
| 1 | Altıntaş | The effect of idiopathic premature ventricular complexes on left ventricular ejection fraction | 1) consecutive patients with more than 1,000 PVC in 24 hr of Holter monitoring admitted to the cardiology clinics | 1) Patients with less than 24 hr and/ or inconclusive Holter recording, coronary artery disease (CAD), his‐ tory of cardiac arrest, sustained ventricular tachycardia (VT), intra‐ cardiac defibrillator (ICD), second‐ or third‐degree AV block, sick sinus syndrome, permanent cardiac pacemaker, known or suspected etiology of cardiomyopathies including, ischemic, restrictive, hypertrophic, diabetic, arrhythmogenic, and non‐compaction before or on admission, genetic cardiac channelopathies, pericardial disease, myocarditis, all forms of atrial fibrillation, thyroid disorders, ane‐ mia, electrolyte disorders, chronic pulmonary disease, pulmonary hypertension, moderate‐to‐severe valvular heart disease, or pulmonary embolism | Yes | LVEF<50% and no structural heart disease | Yes | Premature ventricular complexes were defined as premature beats with abnormally shaped and prolonged QRS complex (different QRS and T‐wave morphology compared with sinus beat and QRS duration >120 ms) arising from an ectopic focus within ventricles. |
| 2 | Sadron | Premature ventricular contraction-induced cardiomyopathy: Related clinical and electrophysiologic parameters. | 1) Group 1: suspected PVCi-CMP as defined by LVEF <50% and increased LV dimensions potentially caused by frequent isolated PVCs and expected to be regressive after successful elimination of the PVC 2) Group 2 : normal LVEF and normal LV dimensions together with frequent PVCs | 1) patients presenting with frequent nonsustained ventricular tachycardia (burden 41% on Holter monitoring) | Yes | PVCi-CMP as defined by LVEF <50% and increased LV dimensions potentially caused by frequent isolated PVCs and expected to be regressive after successful elimination of the PVC | Yes | PVC morphology was defined as right bundle branch block pattern if QRS was positive in lead V1 or left bundle branch block pattern for negative QRS in V1. PVC axis was defined as left inferior (between 01 and 901), left superior (-901 to 01), right inferior (901 to 1801), and right superior (180 to -901). PVC burden (ratio between the number of PVCs and the total number of QRS) on 24-hour ambulatory ECG recordings. In order to minimize the day-to-day variation of PVC burden, average values were calculated when several Holter recordings were available before ablation. Presence of sustained bigeminy/trigeminy (defined by a bi/trigeminy burden 45 minute on 24-hour recordings). |
| 3 | Lee | Ventricular Ectopy in the Context of Left Ventricular Systolic Dysfunction: Risk Factors and Outcomes Following Catheter Ablation | 1. Patients who presented for ablation of PVCs or idiopathic Ventricular arrhythmias 2. Patients with pre-existing scar substrate were included if they underwent a focal ablation procedure | Patients undergoing ablation of macro-reentrant scar Ventricular Tachycardia | Yes | Left ventricular systolic dysfunction due to frequent premature ventricular complexes | No |  |
| 4 | Penela | Influence of myocardial scar on the response to frequent premature ventricular complex ablation | 1. Presence of LV systolic dysfunction (LVEF<50%) 2. Baseline PVC burden >10% at baseline 24h holter 3. Receiving optimal medical therapy 4. nformed consent signed | 1. classic contraindication for late gadolinium enhancement cardiac magnetic resonance | No |  | No |  |
| 5 | Park | Risk factor algorithm used to predict frequent premature ventricular contraction-induced cardiomyopathy | 1. frequents PVCs (>10%/24h) on 2 holter space by at least one week 2. holter monitoring with no evidence of addictional atrial or ventricular tachyarrhythmias 3. presence of detailed clinical symptom description in medical records 4. presence of baseline and follow-up ECG and presence of TTE | 1. history of atrial fibrillation, atrial flutter, atrial tachycardia, non-ustained ventricular tachycardia, sustained VT or evidence of any of these arrhythmias by ECG or Holter monitoring 2. history of myocardial infarction, structural heart disease or heart valve replacement/repair | Yes | global LVEF<50% before PVC suppression and by normalization of the LVEF (>50% and improvement by >10% points) after successful PVC suppression with either radiofrequency ablation | No |  |
| 6 | Agarwal | Relation Between Ventricular Premature Complexes and Incident Heart Failure | adult Patient >18 years | 1. Patients with prevalent systolic or diastolic heart failure 2. Patients with arrhythmogenic right ventricular dysplasia, paroxysmal ventricular tachycardia and valvular heart disease 3. Patients with an heart failure diagnosis during the same visit of first diagnosis of PVC were excluded 4.. Patients who were not resident of California | No |  | No |  |
| 7 | Dukes | Ventricular Ectopy as a Predictor of Heart Failure and Death | Patients > 65 years old randomly asigned to 24h ECG | Patients without a normal LVEF, as determined by the baseline echocardiogram or with prevalent congestive heart failure | No |  | No |  |
| 8 | Ban | Electrocardiographic and electrophysiological characteristics of premature ventricular complexes associated with left ventricular dysfunction in patients without structural heart disease | Patients who underwent radiofrequency catheter ablation (RFCA) because of frequent PVCs (PVC burden≥10%/day) refractory to medical therapy | 1. significant structural heart disease, 2. including coronary artery disease, 3. valvular heart disease, 4. congenital heart disease. 5. Patients with spontaneous or inducible sus-tained ventricular tachycardia were also excluded from the study | No |  | No |  |
| 9 | Yokokawa | Impact of QRS duration of frequent premature ventricular complexes on the development of cardiomyopathy | patients with frequents PVCs who were referred for ablation | 1.cardiomyopathy preceding frequents PVCs 2.delayed enhancement in cardiac magnetic resonance imaging 3.coronary artery disease 4.valvular heart disease 5.hypertensive heart disease | Yes | defined as an ejection fraction <50% that normalized after PVC ablation or an ejection fraction of 50-55% that improved by >10% after PVC ablation | No |  |
| 10 | Yokokawa | Relation of symptoms and symptom duration to premature ventricular complex-induced cardiomyopathy | Patients referred for ablation of frequent PVCs | - | Yes | A patient was considered to have PVC-induced cardiomyopathy if there was improvement by >15% or normalization of the LV ejection fraction after an effective ablation procedure. | No |  |
| 11 | Baman | Relationship between burden of premature ventricular complexes and left ventricular function | Patients with frequent PVCs referred for catheter ablation | - | Yes | A patient was defined to have PVC-induced cardiomyopathy if an abnormal ejection fraction improved by at least 15% or normalized (ejection fraction>50%) after an effective ablation procedure. | No |  |
| 12 | Kanei | Frequent premature ventricular complexes originating from the right ventricular outflow tract are associated with left ventricular dysfunction | 1. Patients with frequent PVC (>10/h) 2. Right ventricular outflow tract morphology (RVOT): PVC with left bundle branch morphology and inferior axis 3. Patient who had other morphology of PVC were included if a majority (>80%) were RVOT | 1. sustained supraventricular tachycardia 2. atrial fibrillation 3. pacemaker 4. history of ischemic heart disease 5. structural heart disease 6. LV dysfunction with segmental wall motion abnormality on echocardiography or with other appartent cause of left ventricular dysfunction (alcohol, HIV) | No |  | No |  |
| 13 | Kawamura | Coupling interval dispersion and body mass index are independent predictors of idiopathic premature ventricular complex-induced cardiomyopathy | 1. Patients referred for successful catheter ablation 2. Frequent and symptomatic PVC 3. Only patients who showed an improvement of LVEF after treatment | 1. Structural heart disease (coronary artery disease, valvular heart disease, hypertensive heart disease, congenital heart disease, prior cardiac surgery) 2. Patients who did not present >10 PVC during electrophysiology study 3. Patient who didn't undergo an 24-h Holter before or after ablation | Yes | defined as an LVEF<50% that normalized or clearly improved by >10% after ablation procedure | No |  |
| 14 | Mountantonakis | Reversal of outflow tract ventricular premature depolarizationinduced cardiomyopathy with ablation: Effect of residual arrhythmia burden and preexisting cardiomyopathy on outcome | 1. Patients with frequent ventricular premature depolarization (>5000 per 24h) and left ventricular cardiomyopathy (LVEF<50%) referred for a catheter ablation. 2. Ventricular premature depolarization were required to have ECG characteristics suggestive of outflow tract origin (right or left bundle branch morphology, inferior axis, negative lead aVL) | Active ischemia or prior infarction as cause of cardiomyopathy | No |  | No |  |
| 15 | Olgun | The role of interpolation in PVC-induced cardiomyopathy | 1) patients with frequent PVCs |  | Yes | PVC-induced cardiomyopathy was defined as an abnormal EF that improved by at least 15% or normalized (EF >50%) after an effective ablation procedure. | No |  |
| 16 | Yokokawa | Predictors of left ventricular dysfunction in patients with frequent premature ventricular complexes | 1) patients who were referred for ablation of frequent PVCs |  | Yes | A LV ejection fraction <50% was considered abnormal. | No |  |
| 17 | Blaye-Félice | Premature ventricular beat-induced cardiomyopathy: Characteristics and prognosis factor for recovery after radiofrequency ablation | 1) patients with dilated CM associated with frequent isolated PVB were included |  | No |  | No |  |
| 18 | Yang | Characteristics of high-burden premature ventricular contraction patients | 1) patients with at least one 24- or 48-hour Holter study showing at least 20% PVCs (designated 'high-burden' PVC patients) |  | No |  | Yes | patients with PVCs that comprise at least 20% of all heart beats recorded during Holter monitoring are considered to have a 'high burden' of PVCs |
| 19 | Latchamsetty | Multicenter outcomes for catheter ablation of idiopathic premature ventricular complexes | 1) patients who underwent radiofrequency catheter ablation for frequent idiopathic PVCs 2) Patients with decreased LV ejection fraction or LV dilation without a known cause other than the PVCs were included in the study | 1) Patients with a history of prior infarcts or delayed enhancement identified by cardiac MRI were excluded. | Yes | A baseline ejection fraction <50% was considered to be evidence of a PVC-induced cardiomyopathy | Yes | The origin of PVCs was classified as right ventricular outflow tract (RVOT), aortic cusps, epicardium, or papillary muscles. If PVCs did not originate from any of these locations, they were defined as having 'other' origin. |
| 20 | Voskoboinik | Predictors of adverse outcome in patients with frequent premature ventricular complexes: The ABC-VT risk score. Hear Rhythm E.P. Gerstenfeld, Department of Cardiac Electrophysiology | 1) patients who had an elevated burden of PVCs (average daily burden >5%) | 1) patients with structural heart disease (including fibrosis on cardiac magnetic resonance imaging) were excluded from both validation cohorts | Yes | adverse LV remodeling (LVEF <45% or left ventricular end-diastolic volume index >75 mL/m2) | No |  |
| 21 | Azizi | Clinical predictors of ventricular tachycardia induced cardiomyopathy | 1) patients with IVT, who underwent ablation (VTA) | 1) excluding all patients with structural heart diseases, history of syncope and implanted device | Yes | patients with more than 15% improvement in EF were considered as PVC-CM | No |  |
| 22 | Yamada | Electrocardiographic characteristics for predicting idiopathic right ventricular outflow tract premature ventricular complex-induced cardiomyopathy | 1) patients undergoing catheter ablation of frequent, symptomatic, and drug-refractory RVOT-VAs with electrocardiographic features including a typical LBBB and inferior axis QRS morphology | 1) Structural heart diseases, including ischemic heart disease, valvular heart disease, arrhythmogenic right ventricular dysplasia/cardiomyopathy, or dilated cardiomyopathy diagnosed prior to the occurrence of frequent RVOT PVCs, were excluded by electrocardiography, echocardiography, computed tomography, and/or cardiac magnetic resonance imaging 2) Patients with a Brugada ECG pattern involving the anterior precordial leads or those diagnosed with Brugada syndrome were also excluded | Yes | In the present study, RVOT PVC-induced cardiomyopathy was defined as an LV ejection fraction (LVEF) of < 50% by echocardiography using Simpson's method from two consecutive sinus rhythm beats. | No |  |
| 23 | Hamon | A new combined parameter to predict premature ventricular complexes induced cardiomyopathy: Impact and recognition of epicardial origin | 1) consecutive patients with frequent PVC (&5%), symptomatic or associated with decreased left ventricular ejection fraction (LVEF) <50%, and referred for RFA were included in this study 2) Patients with multiple PVC morphologies were included when a predominant PVC morphology could be identified 3) The study population included patients with documented SHD such as ischemic heart disease, valvular heart disease, hypertensive heart disease, or dilated CMP diagnosed prior to occurrence of frequent PVC. |  | No |  | Yes | frequent PVC (>5%) |
| 24 | Bas | Effect of circadian variability in frequency of premature ventricular complexes on left ventricular function | 1) patients with frequent PVCs referred for catheter ablation | 1) The presence of structural heart disease | Yes | LV ejection fraction of <50% was considered abnormal. A patient was considered to have PVC-induced cardiomyopathy if the abnormal ejection fraction either normalized or increased at least 15% from the initial value. | No |  |
| 25 | Gunda | Consequences of chronic frequent premature atrial contractions: Association with cardiac arrhythmias and cardiac structural changes. |  |  | Yes | LVEF <50% | Yes | PVC burden was defined as the total number of PVCs over the total beats in a period of 14 days. |
| 26 | Del | Characteristics of premature ventricular complexes as correlates of reduced left ventricular systolic function: study of the burden, duration, coupling interval, morphology and site of origin of PVCs. | 1) patients who underwent radiofrequency catheter ablation for frequent and symptomatic PVCs and had no other identified cause for cardiomyopathy |  | Yes | LVEF (<50%) | No |  |

## Supplemental table 4 – Diagnostics conducted

Diagnostics conducted to assess heart failure (HF) or PVCs in all studies. ILR = Implantable loop recorder.

|  |  |  |  |  |  | **Baseline PVC diagnostics** | | | | **FU PVC diagnostics** | | | |
| --- | --- | --- | --- | --- | --- | --- | --- | --- | --- | --- | --- | --- | --- |
| **Study Nr** | **Author** | **Appropriate HF diagnostic conducted at the beginning of the study?** | **Was there a follow-up?** | **Appropriate HF diagnostic conducted during follow-up?** | **Diagnostics conducted to exclude other causes of HF?** | **ECG** | **Holter** | **ILR** | **Unknown** | **ECG** | **Holter** | **ILR** | **Unknown** |
| 1 | Altıntaş | Yes | No |  | Yes | Yes | Yes | No | No | No | No | No | No |
| 2 | Sadron | Yes | Yes | Yes | Yes | No | Yes | No | No | No | Yes | No | No |
| 3 | Lee | Yes | Yes | Yes | No | No | Yes | No | No | No | Yes | No | No |
| 4 | Penela | Yes | Yes | Yes | Yes | Yes | Yes | No | No | No | Yes | No | No |
| 5 | Park | Yes | No |  | Unclear | Yes | Yes | No | No | No | No | No | No |
| 6 | Agarwal | Unclear | Yes | Unclear | Unclear | No | No | No | Yes | No | No | No | Yes |
| 7 | Dukes | Yes | Yes | Yes | No | No | Yes | No | No | No | No | No | Yes |
| 8 | Ban | Yes | Yes | No | Yes | No | Yes | No | No | No | No | No | Yes |
| 9 | Yokokawa | Yes | Yes | Yes | Yes | Yes | Yes | No | No | Yes | Yes | No | No |
| 10 | Yokokawa | Yes | Yes | Yes | Yes | Yes | Yes | No | No | No | Yes | No | No |
| 11 | Baman | Yes | Yes | Yes | Yes | No | Yes | No | No | No | Yes | No | No |
| 12 | Kanei | Yes | No |  | Unclear | No | Yes | No | No | No | No | No | No |
| 13 | Kawamura | Yes | Yes | Yes | No | Yes | Yes | No | No | No | Yes | No | No |
| 14 | Mountantonakis | Yes | Yes | Yes | No | Yes | Yes | No | No | No | Yes | No | No |
| 15 | Olgun | Yes | Yes | Yes | Unclear | Yes | Yes | No | No | Yes | No | No | No |
| 16 | Yokokawa | Yes | No |  | Unclear | No | Yes | No | No | No | No | No | No |
| 17 | Blaye-Félice | Unclear | Yes | Unclear | Unclear | No | No | No | Yes | No | No | No | Yes |
| 18 | Yang | Yes | Yes | Yes | Yes | No | Yes | No | No | Yes | No | No | No |
| 19 | Latchamsetty | Yes | Yes | Yes | Yes | No | Yes | No | No | Yes | Yes | No | No |
| 20 | Voskoboinik | Yes | Yes | Yes | Yes | Yes | No | No | No | Yes | No | No | No |
| 21 | Azizi | Unclear | No |  | Yes | No | No | No | Yes | No | No | No | No |
| 22 | Yamada | Yes | Yes | Yes | Yes | Yes | Yes | No | No | Yes | Yes | No | No |
| 23 | Hamon | Yes | Yes | Yes | Yes | No | Yes | No | No | No | Yes | No | No |
| 24 | Bas | Unclear | Yes | Unclear | Yes | No | Yes | No | No | No | Yes | No | No |
| 25 | Gunda | Unclear | No |  | Unclear | No | Yes | No | No | No | No | No | No |
| 26 | Del | Yes | Yes | Unclear | Unclear | Yes | Yes | No | No | Yes | No | No | No |

## Supplemental table 5 – Details of the conducted diagnostics in each study

| **Study Nr** | **Author** | **Appropriate HF diagnostic conducted at the beginning of the study?** | **HF Diagnostics at baseline : Describe** | **Follow-up?** | **Appropriate HF diagnostic conducted during follow-up?** | **HF Diagnostics at follow-up : Describe** | **Diagnostics conducted to exclude other causes of HF?** | **Exclusion of other HF causes : Diagnostics** |
| --- | --- | --- | --- | --- | --- | --- | --- | --- |
| 1 | Altıntaş | Yes | Echocardiography | No |  |  | Yes | Unknown |
| 2 | Sadron | Yes | Echocardiography | Yes | Yes | Echocardiography | Yes | Standard evaluation including echocardiography, coronary arteriography, and/or radionuclide angiography. |
| 3 | Lee | Yes | Transthoracic echocardiogram 24-hour Holter monitor | Yes | Yes | Transthoracic echocardiogram 24-hour Holter monitor | No |  |
| 4 | Penela | Yes | Quantification of BNP 12 lead ECG Echocardiography 24h Holter Contrast-enhanced cardiac magnetic resonance | Yes | Yes | functional evaluation Quantification of BNP Echocardiography 24 h holter | Yes | coronary angiography or stress test |
| 5 | Park | Yes | TTE | No |  |  | Unclear |  |
| 6 | Agarwal | Unclear |  | Yes | Unclear |  | Unclear |  |
| 7 | Dukes | Yes | Echocardiogram | Yes | Yes | Echocardiogram was performed 5 years after enrollment (only 842 patients underwent this control) | No |  |
| 8 | Ban | Yes | Two-dimensional echocardiography | Yes | No |  | Yes | exercise ECG |
| 9 | Yokokawa | Yes | echocardiography: LVEF was quantitated by Simpsons formula | Yes | Yes | Echocardiography 3-4 months postablation | Yes | stress testing cardiac magnetic resonance imaging |
| 10 | Yokokawa | Yes | echocardiography | Yes | Yes | Echo at 3 to 6 month post ablation if baselin LEVF had been abnormal | Yes | Cardia magnetic resonance imaging cardiac catheterization stress testing |
| 11 | Baman | Yes | Echocardiography was performed and the LV ejection fraction was assessed based on 2 consecutive sinus rhythm beats using the Simpson formula. | Yes | Yes | Echocardiography was re-peated a mean of 3.7+/- 4.5 months after the ablation proce-dure. | Yes | Coronary artery disease was ruled out by cardiac catheterization or stress testing |
| 12 | Kanei | Yes | echocardiography or gated SPECT | No |  |  | Unclear |  |
| 13 | Kawamura | Yes | Echocardiography: LVEF was calculated by Simpson's method | Yes | Yes | Echocardiography within 6 months post ablation to patients with LV dysfunction | No |  |
| 14 | Mountantonakis | Yes | transthoracic echocardiography | Yes | Yes | transthoracic echocardiography | No |  |
| 15 | Olgun | Yes | Echocardiography | Yes | Yes | Echocardiography | Unclear |  |
| 16 | Yokokawa | Yes | transthoracic echocardiograms | No |  |  | Unclear |  |
| 17 | Blaye-Félice | Unclear |  | Yes | Unclear |  | Unclear |  |
| 18 | Yang | Yes | at least one 24- or 48-hour Holter study | Yes | Yes | 12-lead ECG | Yes |  |
| 19 | Latchamsetty | Yes | echocardiogram, exercise stress testing, cardiac catheterization, and/or cardiac magnetic resonance imaging (MRI) | Yes | Yes | ECG, Holter monitoring | Yes | The presence of structural heart disease was evaluated by echocardiogram, exercise stress testing, cardiac catheterization, and/or cardiac magnetic resonance imaging (MRI) |
| 20 | Voskoboinik | Yes | 14-day ECG patch monitoring, transthoracic echocardiography | Yes | Yes |  | Yes | cardiac magnetic resonance imaging |
| 21 | Azizi | Unclear |  | No |  |  | Yes |  |
| 22 | Yamada | Yes | 12-lead ECG and 24-h Holter monitoring | Yes | Yes | 12- lead ECGs and 24-h Holter monitoring | Yes | electrocardiography, echocardiography, computed tomography, and/or cardiac magnetic resonance imaging |
| 23 | Hamon | Yes | ECG-Holter monitoring and TTE | Yes | Yes | ECG-Holter monitoring and TTE | Yes |  |
| 24 | Bas | Unclear |  | Yes | Unclear |  | Yes | echocardiography, cardiac magnetic resonance imaging, cardiac catheterization, and/or stress testing |
| 25 | Gunda | Unclear |  | No |  |  | Unclear |  |
| 26 | Del | Yes | 2-dimensional transthoracic echocardiography | Yes | Unclear |  | Unclear |  |

## Supplemental table 6 – Assessment of risk factors

Assessment of risk factors for which a quantitative estimate was provided throughout the studies.

| **Risk factors assessed** | **Summarized outcome** | **Uni- vs Multivariable** | **Study ID** |
| --- | --- | --- | --- |
| PVC burden | LVEF (continuous) | Multivar | 1, 18, 26 |
| PVC burden | LVEF change | Univar | 2, 3, 4, 5, 7, 11, 15, 20, 22, 24, 25 |
| PVC burden | LVEF change | Multivar | 2, 3, 5, 6, 7, 8, 9, 10, 11, 13, 15, 16, 17, 19, 20, 21, 22, 23, 24, 25 |
| PVC type: Duration | LVEF (continuous) | Multivar | 1, 26 |
| PVC type: Duration | LVEF change | Univar | 3, 4, 5, 20, 24 |
| PVC type: Duration | LVEF change | Multivar | 3, 5, 23, 24 |
| PVC type: Morphology | LVEF (continuous) | Multivar | 1, 26 |
| PVC type: Morphology | LVEF change | Univar | 2, 4, 11, 14, 20, 24 |
| PVC type: Morphology | LVEF change | Multivar | 17, 19, 24 |
| PVC type: Origin | LVEF change | Univar | 2, 4, 5, 11, 20 |
| PVC type: Origin | LVEF change | Multivar | 2, 5, 9, 17, 19, 23 |
| age | LVEF (continuous) | Multivar | 1 |
| age | LVEF change | Univar | 2, 3, 4, 14, 20 |
| age | LVEF change | Multivar | 6, 13, 19 |
| EF | LVEF change | Univar | 4, 14 |
| EF | LVEF change | Multivar | 14 |
| Sex | LVEF (continuous) | Multivar | 1 |
| Sex | LVEF change | Univar | 2, 3, 4, 5, 11, 20, 24 |
| Sex | LVEF change | Multivar | 3, 5, 6, 9, 19, 23, 24 |
| Coupling interval | LVEF (continuous) | Multivar | 1 |
| Coupling interval | LVEF change | Univar | 2, 3, 20, 22 |
| Coupling interval | LVEF change | Multivar | 13, 20, 23 |
| CAD | LVEF change | Multivar | 6, 19 |
| CAD | LVEF change | Univar | 20 |
| Non sustained VT | LVEF (continuous) | Multivar | 26 |
| Non sustained VT | LVEF change | Multivar | 8, 12, 20 |
| Non sustained VT | LVEF change | Univar | 11, 20, 22 |
| QRS duration | LVEF (continuous) | Multivar | 1, 18 |
| QRS duration | LVEF change | Univar | 2, 5, 22 |
| QRS duration | LVEF change | Multivar | 2, 9, 13, 22, 23 |
| HTN | LVEF change | Multivar | 6, 19 |
| HTN | LVEF change | Univar | 20 |
| Symptoms | LVEF change | Univar | 3, 24 |
| Symptoms | LVEF change | Multivar | 10, 19, 24 |
| SHD | LVEF change | Univar | 4, 14 |
| SHD | LVEF change | Multivar | 14, 23 |
| Palpitations | LVEF (continuous) | Multivar | 26 |
| Palpitations | LVEF change | Univar | 2 |
| Palpitations | LVEF change | Multivar | 2, 23 |
| Interpolation | LVEF (continuous) | Multivar | 1 |
| Interpolation | LVEF change | Univar | 15, 22, 24 |
| Interpolation | LVEF change | Multivar | 15, 23, 24 |
| PVC type: Outflow Origin | LVEF (continuous) | Multivar | 1 |
| PVC type: Outflow Origin | LVEF change | Univar | 3, 11 |
| PVC type: Outflow Origin | LVEF change | Multivar | 3, 17, 19 |

## Supplemental Figure 1 1-4 – Meta-analytic summaries for further, less common risk factors

Supplement Figure 1. 1 : Random effects model of the Coupling interval.: TE -estimate of treatment effect; seTE -standard error of treatment estimate; OR- odds ratio; CI -Confidence interval.

Supplement Figure 1. 2 : Random effects model of polymorphic PVCs. TE -estimate of treatment effect; seTE -standard error of treatment estimate; OR- odds ratio; CI -Confidence interval.

Supplement Figure 1. 3 : Random effects of Outflow tract origin of the PVC as mapped during the electrophysiological (EP) study and confirmed through the ablation. TE -estimate of treatment effect; seTE -standard error of treatment estimate; OR- odds ratio; CI -Confidence interval.

Supplement Figure 1.4 : Random effects of QRS duration. TE -estimate of treatment effect; seTE -standard error of treatment estimate; OR- odds ratio; CI -Confidence interval.

Supplement Figure 1. 4 : Random effects model of sex as a potential risk factor for the development of PVC-induced CM. The estimate of treatment effect (TE) and its standard error (seTE) measure the difference in mean risk between the study group and the controls.Abbreviations: TE -estimate of treatment effect; seTE -standard error of treatment estimate; OR- odds ratio; CI -Confidence interval.

## Supplemental figure 2 – Impact of the publication year on the risk of PVC-CM associated with PVC burden.

The year of publication is shown on the X-axis and the difference in treatment effect on the Y-axis. The PVC-CM risk associated with PVC burden decreased of 0.28% (-0.28%, 95%-CI [-1.02%, 0.46%], p=0.462) with each increase in publication year.

## Supplemental figure 3 - Impact of the study quality on the risk of PVC-CM associated with PVC burden.

The study quality as sum of each component of the QUIPS-tool (with high risk of bias = 0 points, moderate risk of bias = 1 point, low risk of bias = 2 points, allowing for a minimum of 0 points and a maximum of 60 points throughout the overall tool) is shown on the X-axis and the difference in treatment effect on the Y-axis. The PVC-CM risk associated with PVC burden increased of 0.09% (95%-CI [-0.13%, 0.31%], p=0.413) with each increase in quality point of the summed QUIPS tool

## References

1. Huizar, J. F., Ellenbogen, K. A., Tan, A. Y. & Kaszala, K. Arrhythmia-Induced Cardiomyopathy: JACC State-of-the-Art Review. *J Am Coll Cardiol* **73**, 2328–2344 (2019).

2. Marcus, G. M. Evaluation and Management of Premature Ventricular Complexes. *Circulation* 1404–1418 (2020)

3. Latchamsetty, R. & Bogun, F. Premature Ventricular Complex–Induced Cardiomyopathy. *JACC Clin Electrophysiol* **5**, 537–550 (2019).

4. Voskoboinik, A. *et al.* Predictors of adverse outcome in patients with frequent premature ventricular complexes: The ABC-VT risk score. *Heart Rhythm* **17**, 1066–1074 (2020).

5. Riley, R. D. *et al.* A guide to systematic review and meta-analysis of prognostic factor studies. *BMJ (Online)* **364**, (2019).

6. Huizar, J. F., Ellenbogen, K. A., Tan, A. Y. & Kaszala, K. Arrhythmia-Induced Cardiomyopathy: JACC State-of-the-Art Review. *J Am Coll Cardiol* **73**, 2328–2344 (2019).

7. Marcus, G. M. Evaluation and Management of Premature Ventricular Complexes. *Circulation* 1404–1418 (2020)

8. Latchamsetty, R. & Bogun, F. Premature Ventricular Complex–Induced Cardiomyopathy. *JACC Clin Electrophysiol* **5**, 537–550 (2019).

9. Greenland, S. & Longnecker, M. P. Methods for trend estimation from summarized dose-response data, with applications to meta-analysis. *Am J Epidemiol* **135**, 1301–1309 (1992).

10. Hartemink, N., Boshuizen, H. C., Nagelkerke, N. J. D., Jacobs, M. A. M. & Van Houwelingen, H. C. Combining risk estimates from observational studies with different exposure cutpoints: A meta-analysis on body mass index and diabetes type 2. *Am J Epidemiol* **163**, 1042–1052 (2006).

11. Pandey, A. *et al.* Continuous dose-response association between sedentary time and risk for cardiovascular disease a meta-analysis. *JAMA Cardiol* **1**, 575–583 (2016).

12. Sattelmair, J. *et al.* Dose response between physical activity and risk of coronary heart disease: A meta-analysis. *Circulation* **124**, 789–795 (2011).

13. Ding, M., Bhupathiraju, S. N., Satija, A., Van Dam, R. M. & Hu, F. B. Long-term coffee consumption and risk of cardiovascular disease: A systematic review and a dose-response meta-analysis of prospective cohort studies. *Circulation* **129**, 643–659 (2014).

14. Park, K.-M., Im, S. I., Park, S.-J., Kim, J. S. & On, Y. K. Risk factor algorithm used to predict frequent premature ventricular contraction-induced cardiomyopathy. *Int J Cardiol* **233**, 37–42 (2017).

15. Crippa, A. & Orsini, N. Multivariate dose-response meta-analysis: The dosresmeta R package. *J Stat Softw* **72**, (2016).

16. Peters, J. L. Comparison of Two Methods to Detect Publication Bias in Meta-analysis. *JAMA* **295**, 676 (2006).

17. DerSimonian, R. & Laird, N. Meta-analysis in clinical trials. *Control Clin Trials* **7**, 177–188 (1986).

18. Weir, C. J. *et al.* Dealing with missing standard deviation and mean values in meta-analysis of continuous outcomes: A systematic review. *BMC Med Res Methodol* **18**, 1–14 (2018).

19. Chinn, S. A simple method for converting an odds ratio to effect size for use in meta-analysis. *Stat Med* **19**, 3127–31 (2000).

20. Nolan, M. T., Russell, D. J., Negishi, K. & Marwick, T. H. Meta-Analysis of Association Between Mediastinal Radiotherapy and Long-Term Heart Failure. *American Journal of Cardiology* **118**, 1685–1691 (2016).

21. di Marco, A. *et al.* Late Gadolinium Enhancement and the Risk for Ventricular Arrhythmias or Sudden Death in Dilated Cardiomyopathy: Systematic Review and Meta-Analysis. *JACC Heart Fail* **5**, 28–38 (2017).

22. Sadron Blaye-Felice, M. *et al.* Premature ventricular contraction-induced cardiomyopathy: Related clinical and  electrophysiologic parameters. *Heart Rhythm* **13**, 103–110 (2016).

23. Yokokawa, M. *et al.* Impact of QRS duration of frequent premature ventricular complexes on the development of cardiomyopathy. *Heart Rhythm* **9**, 1460–1464 (2012).

24. Blaye-Félice Sadron, M. A. *et al.* Premature ventricular beat-induced cardiomyopathy: Characteristics and prognosis factor for recovery after radiofrequency ablation. *Heart Rhythm* **11**, S70–S71 (2014).

25. Latchamsetty, R. *et al.* Multicenter outcomes for catheter ablation of idiopathic premature ventricular complexes. *JACC Clin Electrophysiol* **1**, 116–123 (2015).

26. Hamon, D. *et al.* A new combined parameter to predict premature ventricular complexes induced cardiomyopathy: Impact and recognition of epicardial origin. *J Cardiovasc Electrophysiol* **27**, 709–717 (2016).

27. Olgun, H. *et al.* The role of interpolation in PVC-induced cardiomyopathy. *Heart Rhythm* **8**, 1046–1049 (2011).

28. Katz, L. N., Langendorf, R. & Cole, S. L. An unusual effect of interpolated ventricular premature systoles. *Am Heart J* **28**, 167–176 (1944).

29. Bas, H. D. *et al.* Effect of circadian variability in frequency of premature ventricular complexes on left ventricular function. *Heart Rhythm* **13**, 98–102 (2016).

30. Park, K.-M., Im, S. I., Park, S.-J., Kim, J. S. & On, Y. K. Risk factor algorithm used to predict frequent premature ventricular contraction-induced cardiomyopathy. *Int J Cardiol* **233**, 37–42 (2017).

31. Ban, J.-E. *et al.* Electrocardiographic and electrophysiological characteristics of premature ventricular complexes associated with left ventricular dysfunction in patients without structural heart disease. *Europace* **15**, 735–741 (2013).

32. Kanei, Y. *et al.* Frequent premature ventricular complexes originating from the right ventricular outflow tract are associated with left ventricular dysfunction. *Annals of Noninvasive Electrocardiology* **13**, 81–85 (2008).

33. Voskoboinik, A. *et al.* Predictors of adverse outcome in patients with frequent premature ventricular complexes: The ABC-VT risk score. *Heart Rhythm* **17**, 1066–1074 (2020).

34. Yokokawa, M. *et al.* Relation of symptoms and symptom duration to premature ventricular complex-induced cardiomyopathy. *Heart Rhythm* **9**, 92–95 (2012).

35. Kawamura, M. *et al.* Coupling interval dispersion and body mass index are independent predictors of idiopathic premature ventricular complex-induced cardiomyopathy. *J Cardiovasc Electrophysiol* **25**, 756–762 (2014).

36. Lee, A., Denman, R. & Haqqani, H. M. Ventricular Ectopy in the Context of Left Ventricular Systolic Dysfunction: Risk Factors and Outcomes Following Catheter Ablation. *Heart Lung Circ* **28**, 379–388 (2019).
